# Additive effects of light and branching on fruit size and chemical fruit quality of greenhouse tomatoes

**DOI:** 10.3389/fpls.2023.1221163

**Published:** 2023-10-24

**Authors:** Martina Paponov, Michel J. Verheul, Petre I. Dobrev, Ivan A. Paponov

**Affiliations:** ^1^ Division of Food Production and Society, Norwegian Institute of Bioeconomy Research (NIBIO), Ås, Norway; ^2^ Institute of Experimental Botany, Czech Academy of Sciences, Prague, Czechia; ^3^ Department of Food Science, Aarhus University, Aarhus, Denmark

**Keywords:** tomato, light-emitting diode, fruit growth, fruit quality, plant hormones, sink-source relationship, branching

## Abstract

**Introduction:**

Greenhouse tomato growers face the challenge of balancing fruit size and chemical quality traits. This study focused on elucidating the interplay between plant branching and light management on these traits, while maintaining consistent shoot density.

**Methods:**

We evaluated one- and two-shoot plants under varying top light intensities using high-pressure sodium lamps and light-emitting diode (LED) inter-lighting.

**Results:**

The reduced yield in the two-shoot plants was mainly due to smaller fruit size, but not due to source strength limitations, as evaluated through leaf weight ratio (LWR), chlorophyll index, specific leaf area (SLA), leaf dry matter percentage, and stem soluble carbohydrate accumulation. Enhanced lighting improved fruit weight and various fruit traits, such as dry matter content, total soluble carbohydrate content, and phenolic content, for both one- and two-shoot plant types. Despite lower mean fruit weight, two-shoot plants exhibited higher values for chemical fruit quality traits, indicating that the fruit growth of two-shoot plants is not limited by the available carbohydrates (source strength), but by the fruit sink strength. Diurnal analysis of fruit growth showed that two-shoot plants had reduced expansion during light transitions. This drop in fruit expansion was not related to changes in root pressure (measured as xylem sap exudation from decapitated plants), but might be related to diminished xylem area in the stem joint of the two-shoot plants. The concentration of several hormones, including cytokinins, was lower in two-shoot plants, suggesting a reduced fruit sink capacity.

**Discussion:**

The predominant impact of branching to two-shoot plants on sink capacity suggests that the fruit growth is not limited by available carbohydrates (source strength). Alongside the observation that light supplementation and branching exert independent additive effects on fruit size and chemical traits, this illuminates the potential to independently regulate these aspects in greenhouse tomato production.

## Introduction

Tomato is the most economically important horticultural crop and is widely used as a model plant, particularly for investigations of fruit development and fruit quality. The high market value and challenges imposed in transporting tender tomato fruits have made tomato the main income crop for local greenhouse production. The optimization of climate control, crop management, and tomato breeding for greenhouse production has doubled the yield during the last decades [reviewed in (Bertin and Genard, 2018)]; however, this high yield is often associated with poor fruit quality, which lowers the customers’ interest in greenhouse-cultivated tomatoes (Rana et al., 2014). Thus, understanding the physiological processes that can improve yield and fruit quality is a key element for future success in greenhouse tomato production.

One of the main reasons for the reduction in yield and fruit quality of greenhouse-grown tomatoes is low light intensity (Gruda, 2005), especially in northern latitudes or during the winter season. To overcome low light conditions, supplemental lighting is widely applied in greenhouses (Marcelis et al., 2006). Traditionally, the light is supplied from the top of the greenhouse, mostly from high-pressure sodium (HPS) lamps. The introduction of cost-efficient LED lamps with low heat emission provided the possibility of using LED inter-lighting with a more uniform light distribution along the canopy (Gomez et al., 2013). The implementation of uniform lighting has a dual purpose: firstly, it prevents the top canopy leaves from receiving excessive illumination, and secondly, it promotes an increase in photosynthetic activity in the lower leaves (Evans et al., 1993). Inter-lighting also provides sufficient light to the otherwise shaded lower leaves to maintain a good photosynthetic rate and prevent leaf senescence (Davis and Burns, 2016; Bantis et al., 2018). The combination of supplemental top-lighting and LED inter-lighting increases the yield of tomato plants, with increased fruit weight being a commercially important component of this yield enhancement (Cockshull et al., 1992; Li et al., 2015; Paponov et al., 2020).

The simplest mechanism that explains the positive effect of additional light on fruit weight is related to a direct effect of light on source activity (i.e., plant photosynthesis), as increased photosynthetic activity can increase carbohydrate transport to the fruits (Lemoine et al., 2013). The enhanced supply of carbohydrates can affect fruit size through increased cell division, which is typically completed within 10 days after anthesis (Ho and Hewitt, 1986), and by cell enlargement during the subsequent fruit developing stage (Ho, 1996). During the cell division stage, sugar can act as a signal that stimulates cell division, thereby defining a greater sink capacity of the fruit (Palmer et al., 2015). During the cell enlargement phase, an enhanced supply of carbohydrates generates a higher turgor pressure, which stimulates cell elongation and results in a heavier fruit weight (Kanayama, 2017). Thus, the ultimate fruit weight depends on the dynamic availability of carbohydrates to individual fruits at different stages of fruit development.

Apart from the supply from source activity, the carbohydrates available to individual fruits depend on the competition for resources among the plant’s trusses (inflorescences) and between fruits within one truss (Bertin, 1995). Tomato plants have a complex bicollateral phloem system that allows for an unusual transport in both directions, where basal leaves export photosynthate to the upper stem and shoot apex, while upper leaves export to the lower stem and roots (Khan and Sagar, 1966; Hocking and Steer, 1994). This pattern of assimilate movement is considered “inefficient” (Ryle et al., 1981), because it goes against the principle of using the shortest translocation pathway between a source and sink. However, this movement pattern can help buffer the strong light gradient along the canopy and provide sufficient assimilates for key organs (e.g., roots and fruits during the loading stage) that are localized far from the upper leaves exposed to the highest light intensity. Thus, for tomato plants, inter-lighting might have a positive effect due to enhanced total photosynthesis, rather than from a better assimilate supply for organs located distantly from the upper leaves.

Fruits within a truss compete for resources, leading to smaller distal fruits compared to proximal ones. Under conditions of unlimited carbohydrate supply, the size differences between distal and proximal fruits are mainly defined during the cell division stage. At the end of that stage, the ultimate cell number is related to the sink strength (Bangerth and Ho, 1984). However, source limitation causes a competition among fruits during both the cell division and fruit loading stages and creates a greater weight deviation between distal and proximal fruits (Paponov et al., 2020). Thus, the relative difference observed between distal and proximal fruit weights can be used to characterize a source limitation and/or an imbalance between source and sink activities and should be increased when the source activity of plants is decreased.

Supplemental lighting increases the fruit size while also promoting the accumulation of primary metabolites, such as sugars and secondary compounds, in the fruits, thereby improving several fruit quality traits. For example, supplemental light increases the content of soluble sugars (Davies and Hobson, 1981), ascorbic acid (Dumas et al., 2003), and phenolics (Hernandez et al., 2019) but does not appear to change organic acid concentrations (Dorais et al., 2001). The main mechanism involved in the enhanced accumulation of secondary compounds might be related to the increased amount of C-skeletons available for the biosynthesis of secondary compounds (Løvdal et al., 2010). In addition, LED inter-lighting can have different effects than top lighting on fruit quality, given that direct exposure of the fruits themselves to light can have a strong effect on the accumulation of several secondary compounds (Gautier et al., 2009; Pek et al., 2011) and that the accumulation of primary and secondary compounds shows specific responses to the light spectrum (Ntagkas et al., 2020). Nevertheless, despite numerous investigations on the effect of light on fruit quality, the interplay between light intensity, distribution, and cultivation techniques on yield and quality remains underexplored.

One widely used cultivation technique, which can increase fruit quality (e.g., enhancing soluble solids content) is to grow tomato plants with two shoots connected to a single root system. However, although this cultivation technique improves quality, it also reduces fruit size (Max et al., 2016; Vargas et al., 2017). This trade-off between fruit size and fruit quality is commonly observed (Hanson et al., 2004), but its underlying mechanism remains elusive.

Tomato plant cultivation as one- or two-shoot plants has been widely used as a tool to study long-distance interactions between plant roots and shoots and the involvement of plant hormones, such as auxin and cytokinins (Li and Bangerth, 1999; Li and Bangerth, 2003; Kotov and Kotova, 2018). These studies showed that auxin is predominantly synthesized in the shoot (mostly in young tissues, such as shoot apical meristems) and transported to the roots, suppressing cytokinin production. This results in decreased cytokinin concentrations in the xylem sap (Bangerth, 1994; Li et al., 1995; Kotov and Kotova, 2018). While the two-shoot model has been explored in young plants of various species, its applicability to older plants during the generative stage, and its influence on yield and fruit quality, warrants further investigation.

In two-shoot plants, each shoot shares the root’s capacity, necessitating double the root activity or transport efficiency to sustain the same solute flux per shoot as in one-shoot plants. The xylem water supply is an important component of fruit growth, whereas treatments that decrease root activity (root pressure), such as drought and salt, usually enhance fruit quality traits such as total soluble solids and soluble sugars (Araki et al., 2001; Van de Wal et al., 2017; Cui et al., 2020). The beneficial impact of light on these traits is linked to an increased phloem transport relative to xylem transport (Hanssens et al., 2015), further supporting that the balance between these transports contributes substantially to fruit quality traits. As two-shoot plants have only one root system for both shoots, we hypothesize that the effect of two-shoot plants on fruit quality traits may occur through modulation of the balance between xylem and phloem transport into the fruits.

The aim of the present work was to understand the mechanisms underlying the effects of plant branching and their interplay with different light distributions on fruit weight and fruit quality. We assumed that if the mechanisms of action between branching and modified light supply are independent, no significant interactions would be noted between the branching and light levels (i.e., top light or LED inter-lighting). In addition, we asked whether two-shoot plants show decreased xylem transport per shoot and a consequent change in the phloem/xylem balance that could contribute to better fruit quality. Ultimately, we tested the hypothesis that the number of shoots on the plants, in combination with different light conditions, modulates the xylem sap plant hormone composition, and especially the concentration of cytokinins, as these are key players in the sink activity of tomato plants during the generative period.

## Materials and methods

The investigations were conducted simultaneously in two identical, structurally modern, and adjacent greenhouse compartments at the NIBIO Særheim research station, located in southwestern Norway (lat. 58.47 long. 5.41, alt. about 90 m a.s.l.) from 29 July 2018 until 15 February 2019. We used the indeterminate variety Dometica (*Solanum lycopersicum*, DOMETICA RZ F1, Rijk Zwaan), a long-cropping type with upright foliage and high production output. The tomato fruits are loosely arranged within the truss. These tomatoes are red, round, and firm and have an average fruit weight of 80 g with good flavor due to their high sugar and acidity content.

### Experimental setup

**Figure 1:**
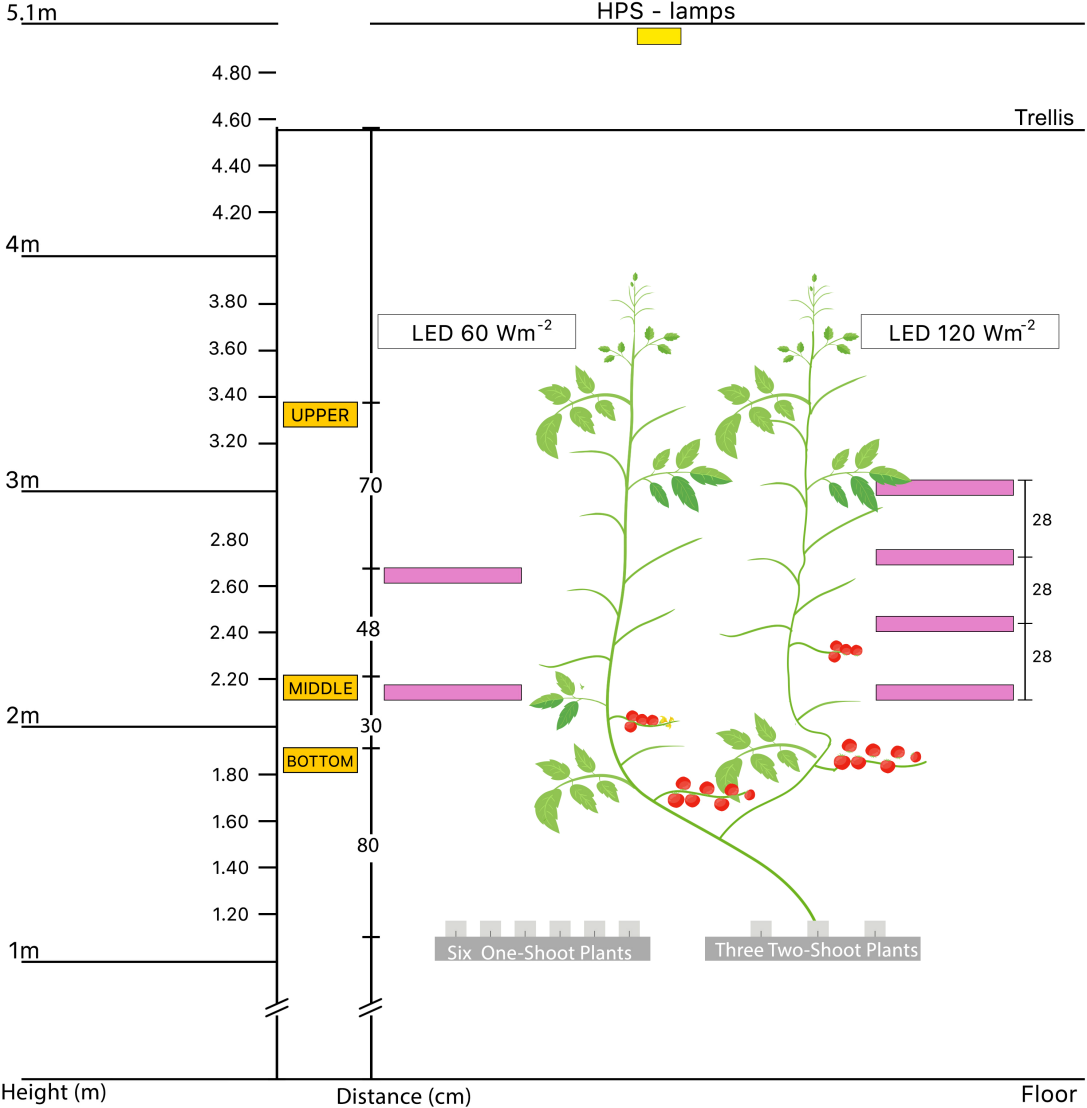
Schematic diagram of the experimental setup used to investigate the effects of different lighting treatments on one-shoot and two-shoot plants. The diagram shows an aggregated representation of all treatments, emphasizing the two-shoot plant and the positions of the top light and LED lamps. Notably, each stem of the two-shoot plants received identical lighting conditions to ensure uniform treatment exposure. The experimental setup consisted of two separate compartments: a low top light at 161 W m^-2^ and a high top light at 242 W m^-2^. Three levels of supplemental LED inter-lighting were used in combination with the top light treatments: without LED, with supplemental 60 W m^-2^ LED inter-lighting, or with 120 W m^-2^ LED inter-lighting (only with the low top light treatment).

The experimental setup included two top light treatments, each in a separate compartment: a low top HPS light at 290 µmol m^-2^ s^-1^ (161 W m^-2^) and a high top HPS light at 436 µmol m^-2^ s^-1^ (242 W m^-2^). HPS top light was combined with three levels of supplemental LED inter-lighting: no LED, supplemental 60 W m^-2^ LED inter-lighting, or 120 W m^-2^ LED inter-lighting (only with the low top light treatment) (**Figure 1**). LED lamps from Union Power Star (160 W, Munich, Germany) were positioned in the middle of the V-row system, emitting light horizontally in two directions. They operate with wavelength bands of 450 and 660 nm at a diode energy ration of 20/80. The detailed light environment was described in our previous publication (Verheul et al., 2022).

For each of these light treatments, we investigated their effects on one-shoot and two-shoot plants. In total, we investigated 10 treatments, 8 of which formed a complete 3-factorial experiment, including the treatments of top light at 161 W m^-2^, top light at 161 W m^-2^ + LED 60 W m^-2^, top light at 242 W m^-2^, and top light at 242 W m^-2^ + LED 60 W m^-2^; each light treatment was applied to one-shoot and two-shoot plants. The additional two treatments with top light at 161 W m^-2^ + LED 120 W m^-2^ for one- and two-shoot plants corresponded to the total light intensities of the high-top light compartment, but the light was provided strongly from the side. To account for potential variation between LED lamps, plants within these treatments were exposed to different LED lamps.

It is important to note that these treatments were deliberately chosen. The environment with 161 W m^-2^ HPS + 60 W m^-2^ LED has approximately the same photosynthetic photon flux density (PPFD) as the environment with 242 W m^-2^ HPS. Similarly, the environment with 161 W m^-2^ HPS + 120 W m^-2^ LED has approximately the same PPFD as the environment with 242 W m^-2^ HPS + 60 W m^-2^ LED.

### Growing conditions

Each compartment was 224 m^2^ (17.5 m × 12.8 m) in size, with 6 gutters with double rows and 2 gutters with simple border rows in each compartment. The distance between the rows was 90 cm, and the distance between gutters was 180 cm. High pressure sodium (HPS) lamps (Philips GP Plus 600 W and 750 W, Gavita Nordic AS, Norway) were positioned ca. 1.5 m above the top of the canopy, at a height of 6 m. One double row was used for each light treatment. Each row included 60 one-shoot plants and 18 two-shoot plants, for a total of 36 shoots. For the purpose of growth and yield analysis, we collected data from 5 replications. Each replication comprised two plants from the one-shoot treatment (equating to two shoots) and one plant from the two-shoot treatment (equating to two shoots). The ambient climate was monitored every 5 min by a Priva Connext horticultural computer, which coordinated all climate, light, irrigation, water, and energy processes after adjustments. Detailed climatic conditions were described in our previous publication (Verheul et al., 2022).

The plants were first raised in a neighboring greenhouse in 0.5 L rockwool cubes (sown 29 July 2018). By 45 days after sowing, when the first flower truss appeared, the plants were transported with the cube to the greenhouse compartments (12 September 2018). The plants were left for 5 days under mild drought stress besides the plant hole to promote better rooting in the cube and to adapt the plants to the new environment. The tomato plants were then transplanted together with the cube onto holes in standard rockwool slabs (90 cm × 10 cm × 15 cm) placed with a distance of one slab per 100 cm on gutters at 110 cm height from the ground. The plants were kept under drought (given 133 mL nutrient solution 3–4 times per day per shoot at 8:00, 11:30, and 17:00). Excess apical and basal leaves and suckers were removed regularly to promote generative growth. The tomato plants were trained to one-shoot plants by removing all suckers or to two-shoot plants by saving the side shoot in the leaf axis just below the first flower truss. Six one-shoot plants or 3 two-shoot plants were planted on each rockwool slab. Each shoot was supported by a twine strand wrapped around the plant at the base and fixed on the overhead trellis system. Of the six one-shoot plants on a slab, half were secured to one side and the other half to the opposite side of the row, arranged in an alternating sequence. Meanwhile, each shoot of the two-shoot plants was attached to one side to form a high wire culture in a V-row system (Peeters and Welles, 2005). The final plant density was 3 plants/m^2^ and 3 shoots/m^2^ for the one-shoot and two-shoot plants, respectively. The plants were regularly maintained by removing all excess suckers and leaves and all leaves beneath the latest harvested tomato truss. The fruit trusses were pruned to seven fruits per truss just after the fruit set of each truss.

During the establishing phase of six weeks after transplanting, plants were grown under sunlight and at a maximum of 12 h of HPS lamp light to avoid excessive assimilate production. The CO_2_ concentration was kept at 600 ppm for the first 13 weeks of the experiment, until week 50 (for 2018).

Once the plant tops reached approximately 150 cm above the rockwool cubes, we installed the LED lamps. For the 60 W m^−2^ LED treatments, lamps were installed between the shoots at heights of 110 cm and 158 cm above the rockwool cubes. For 120 W m^-2^ LED inter-lighting, lamps were set at 110, 138, 166, and 194 cm heights above the rockwool blocks (**Figure 1**).

### Regulation of climatic conditions and irrigation when plants reached steady state

From week 44–45 (starting 29 October 2018–03 December 2018), 6 weeks after final transplanting, the tomato plants reached a size of about 250 cm. The plant reached a ‘steady state condition’, when the first tomato turned red and a stable balance was established between developing plant parts and ripening of the tomatoes.

Good plant vigor in each compartment with different top light preconditions was maintained by adjusting the temperature according to the stem diameter approximately 25 cm below the tomato shoot apex. The stem diameter is a sensitive indicator of plant resource distribution, as it indicates investments in vegetative or generative growth. Based on our own in-house experiences and others (Mireille et al., 1997), the best tomato yield is obtained when the diameter of the stem is between 10 and 12 mm. The stem diameter was measured once per week for the control treatments, which consisted of plants that did not receive supplemental LED lighting.

When plants reached the steady state, the climate settings became stable. The artificial lighting (HPS lamps and LED lamps) were switched on for 18 h from 06:15–00:15, as natural incoming light had no impact in the wintertime. The temperature set points were slightly adjusted on a weekly basis based on plant vigor measurements. The detail temperature conditions are presented in the previous publication (Verheul et al., 2022). Humidity was maintained between at about 63% and 75% for the low and high top light compartment.

From mid-December, the setpoint for CO_2_ concentration was 1000 ppm, which resulted in about 900 ppm CO_2_ in the greenhouse. When the windows were opened for humidity release and temperature control, the CO_2_ concentration was kept at 600 ppm. In the winter, during our investigations, the windows were mostly closed. The plants were drip irrigated with a complete nutrient solution based on standardized recommendations and containing the following: 26.43 mM NO_3_^-^, 1.68 mM NH_4_^+^, 2.23 mM P, 8.72 mM K, 10.63 mM Ca, 2.71 mM Mg, 2.67 mM S, 0.3 mM Na, 0.1 mM Cl, and micronutrients with the following concentrations: 63 μmol Fe, 27 μmol Mn, 10 μmol Zn, 68 μmol B, 6 μmol Cu and 1.6 μmol Mo. The electrical conductivity of the nutrient solution was 3.6 mS cm^-1^, the pH was 5.9, and the daily drainage percentage was 30%. Irrigation and drainage were registered continuously using a weighing scale (Priva GroScale) combined with a drainage sensor. Plants were irrigated for 3-4 min (33 mL/min) at 8:00, 9:00, 12:00, 14:00, and 15:30. If the drainage percentage was below 30%, an additional irrigation was performed, but not later than 17:00. Conventional heating pipes provided heating in the compartments.

Tomato plants were grown under HPS top light with an installed capacity of 161 W m^-2^ and 242 W m^-2^. The photosynthetically active radiation (PAR) 30 cm above the apex of the plants in each light treatment was 180, 214, 202 and 435, 458 μmol m^-2^ s^-1^ for low top light with no LED, + LED 60 W m^-2^,+ LED 120 W m^-2^, high top light with no LED, and high top light +LED 60 W m^-2^, respectively. The results are based on 10 measurements with a zenith direction of PAR sensor under complete exclusion of sunlight.

### Plant care and tomato harvest

The tomato flowers were pollinated by bumblebees, and pollination success was checked two times per week. On a weekly basis, the plants were lowered by about 30 cm, all side shoots and three leaves below the truss on which fruits were reaching the turning stage (Grierson and Kader, 1986) were removed, and trusses were pruned to seven fruits per truss just after fruit set. Tomato fruits were harvested two times per week for all treatments. The harvested fruits were weighed individually, and the fruit position in the truss and number of trusses were recorded for 10 plants per treatment during the growing period. The first day of harvest was 6 November 2018. The tomato fruit next to the stem was designated as position one and the distal tomato position was designated as position 7.

### Determination of specific leaf area

The fresh weights of 10 proximal leaflets were taken at three height levels for all treatments. The “upper position” represented a leaflet sample from a fully developed leaf about 40-55 cm below the apex, the “middle position” was a leaflet sampled at the height of the lower LED lamp (110 cm above the rockwool cube), and the “bottom position” was a leaflet sampled from the oldest leaf of the plant. The leaf area was determined using an LI-3100 Area Meter (LI-COR, inc. Lincoln, Nebraska, USA). The leaves were dried at 65°C for 48 h and weighed.

### The final harvest

The plant yield was measured by including a destructive harvesting over a period of 5 days (11–15 February 2019), where 2 plants per treatment were removed from the canopy in a random order. The final length of the plant (cm), fresh weight of the stem, number of nodes and remaining leaves, fresh weight of leaves, and the total number of trusses with ripe tomatoes were recorded. Dry matter % (DM) of the fruits at different developmental stages was determined by measuring the fresh weight (FW) of the tomato fruits at position 3 from the basal end of a truss containing 7 fruits. This was carried out separately for the 5 lowest trusses, while the green tomatoes of the younger trusses (6–12) were collected together and measured as a group. All samples, including tomatoes, stem(s), and all leaves per plant of all treatments (n=10), were dried at 70°C until complete dryness. Since the branches of two-shoot plants have different length, we calculated average length of the entire plant as the mean of the lengths of both branches. Even though we trained the side branch below the first truss of two-shoot plants, the side shoot appeared to develop fewer trusses. To allow comparison of the data for one- and two-shoot treatments, we calculated number of red fruits/shoot.

### Fruit quality analysis

For quality assessment, fruit samples were collected on 6 days (05 December 2018, 17 December 2018, and 07 January 2019) for one-shoot plants and (06 December 2018, 18 December 2018, and 11 January 2019) for two-shoot plants. In total, 9 replications per treatment were investigated; each replication consisted of six fruits of equal size and with a ripeness of grade 8, determined visually based on a color scale from Bama AS, Norway ranging from 1 (green) to 12 (deep red). Each tomato was measured for firmness at three locations on the pericarp on a scale from 1 to 100, where 100 means full firmness and 1 a complete lack of firmness (Durofel firmness tester, Agro Technologies, France). One-quarter of each of the six tomatoes was immediately homogenized with a handheld blender on the harvesting day. The resulting homogenate was used to determine the soluble solid content (SSC) measured with a digital PR-101α refractometer (ATAGO, Japan), and the total titratable acidity (TTA), expressed as a percentage of citric acid equivalents (CAE) per 100 g FW measured with the 794 Basic Titrino (Metrohm, Switzerland) with potentiometric detection and a final pH of 8.2. An aliquot of the tomato fruit homogenate was immediately frozen in liquid nitrogen and lyophilized in a freeze drier for 24–48 h.

### Assay of total phenolic content

The total phenolic content was estimated in the tomato homogenates using the Folin–Ciocalteu assay (Ainsworth and Gillespie, 2007). A 20 mg sample of freeze-dried tomato homogenate was extracted with 1.8 mL 0.5% acetic acid in 80% methanol in darkness for 6 h at 25°C with shaking at 400 rpm. The homogenate was then centrifuged for 5 min at 13,000×g (Micro Star 17R, VWR, Radnor, PA, USA) and 100 µL of the supernatant was combined with 200 µL of 10% Folin–Ciocalteu (F–C) reagent (F9252, Merck, Darmstadt, Germany) and vortexed. An 800 µL volume of 700 mM Na_2_CO_3_ solution (S7795, Merck, Darmstadt, Germany) was added, thoroughly mixed, and incubated at room temperature for 2 h in darkness. The samples were centrifuged again to pellet any leftover tomato fragments. Triplicate 200 µL volumes of the supernatant solution were then transferred to a spectrophotometric plate reader (Multiscan GO, Thermo Fisher Scientific, Waltham, MA, USA), and the absorbance was measured for each well at 765 nm at room temperature. Measurements were standardized against gallic acid (48630, Merck, Darmstadt, Germany) (40 μM–1.2 mM in 0.5% acetic acid in 80% MeOH).

### Quantification of glucose, fructose, and sucrose contents in one-shoot and two-shoot stems

The complete dried stem of one-shoot plants and the complete initial and longer shoot for two-shoot plants were chopped up and an aliquot was ground to fine dust in a grinding mill (Star-Beater, VWR, USA). For soluble carbohydrate extraction, 75 mg of stem material was transferred into 5 mL Eppendorf tubes and extracted 3 times with 1.6 mL 80% ethanol for 15 mins at 80°C, followed by centrifugation at 3000 × g for 10 mins after each extraction. The collected supernatants were combined in a 5 mL Eppendorf tube, brought to a 5 mL volume with 80% ethanol, and 60 mg of finely ground activated charcoal was added to each tube. The tubes were closed, shaken briefly by hand, left to stand for 5 min, and then centrifuged at 3000 × g for 15 min to obtain a clear extract.

The glucose, fructose, and sucrose contents were quantified using sequential enzymatic assays with photometric detection in a spectrophotometric plate reader (Multiscan GO, Thermo Scientific) according to (Zhao et al., 2010). Glucose concentrations were determined by transferring three 20 µL aliquots of each extracted sample into separate wells of a 96-well UV-Star microplate (Greiner). The microplate, without standards added, was placed into an oven at 50°C to dry for 60 min. The dried material was then resuspended by the addition of 20 μL of deionized water. For the calibration curve, 20 µL of a standard glucose solution (0, 0.005, 0.0125, 0.025, 0.050, 0.125, 0.25, to 0.5 mg mL^−1^ in DI water, prepared weekly) was added in triplicate to each microplate into the remaining wells. The glucose hexokinase (HK) assay reagent (G3293, Supelco) was added into each well (100 µL per well) according to the manufacturer’s instructions. The 96-well plate (UV-STAR, Greiner Bio-One) was covered with a lid and incubated inside the plate reader for 15 min at 30°C. The absorbance of samples, blanks, and standards was measured at 340 nm at 30°C and precision mode. The amount of fructose was determined with the phosphoglucose isomerase (PGI) assay by adding 10 μL of PGI assay reagent (0.2 M HEPES with pH 7.8) to each well previously used for glucose quantification. The absorption was measured at 340 nm after incubation inside the spectrophotometer for 15 min at 30°C. The sucrose amount was determined by adding 10 μL of invertase assay reagent (10 mg mL^-1^, I4504, 300 units/mg, Sigma) in 0.1 M Na-citrate buffer pH 6.0 to each well. The absorption was measured at 340 nm after incubation inside the spectrophotometer for 60 min at 30°C. Glucose, fructose, and sucrose absorption values were calculated based on triplicate replications and used in statistical analysis, as suggested by (Zhao et al., 2010).

### Chlorophyll index

The chlorophyll index was measured (Hansatech Instruments Chlorophyll Content System CL-01, Norfolk, United Kingdom) on each of the registered plants at three different heights (upper, middle, and bottom) at 2 different days (20 November 2018 and 9 January 2019). The second distal leaflet pair was measured 3 times and the average values were used for statistical analysis The “upper” position included the first fully developed leaf (which is still expanding) approximately 6 or 7 leaves counted from the top of the plant, the “middle” position was at the level of height of the lower LED lamp, and the “bottom” position was the lowest leaf.

### Daily fruit growth

The diurnal changes in fruit diameter were measured over 2–4 days on the third tomato fruits in a truss with fruits with diameters of 2.6–3.4 cm; these fruits were typically located about 40–50 cm below the tops of the plants. The change in fruit diameter was monitored using the fruit and vegetable dendrometer from Ecomatik and the Dendrometer Data Logger from Ecomatik (DL18, Dachau/Munich, Germany) interfaced to a 4-channel analog Dendrometer Data Logger from HOBO (Onset Computer Corporation, Bourne, USA). The experiment was replicated at least twice so that at least 9 and 5 fruits were investigated for control plants (161 W m^-2^ HPS lighting) for one-shoot and two-shoot plants, respectively. The values show the relative increase in fruit diameter every 30 min after min–max normalization (Han et al., 2012) with the assumption that the fruit diameter would increase by 1 U over a 24 h period.

### Xylem sap collection and hormone analysis

The xylem sap was sampled by the root pressure method (Alexou and Peuke, 2013) on 5 sequential days on 1 replication per treatment chosen randomly. The plants were cut with a clean garden scissor 5 cm above the root–shoot interface. The cut surface was cleaned with deionized water and a silicon tube was fixed over the stump and sealed with silicone grease. The xylem exudate collection was initiated 30 min later. The sap was collected with a pipette for 30 min, transferred to plastic vials on ice, and subsequently frozen in liquid nitrogen and stored at -80°C. To minimize the potential effect of diurnal variation, we spred the harvesting process over 5 days. This approach ensured that xylem sap collections were consistently performed during a relatively short and uniform period each day, specifically between 10:00 and 11:30. The hormones in the xylem sap were analyzed with an HPLC system (Ultimate 3000, Dionex, Sunnyvale, CA, USA) coupled to a 3200 Q TRAP hybrid triple quadrupole/linear ion trap mass spectrometer (Applied Biosystems, Waltham, MA, USA). The sample preparation and analysis procedures were as described by Paponov et al. (Paponov et al., 2021).

## Results

### Yield

**Figure 2:**
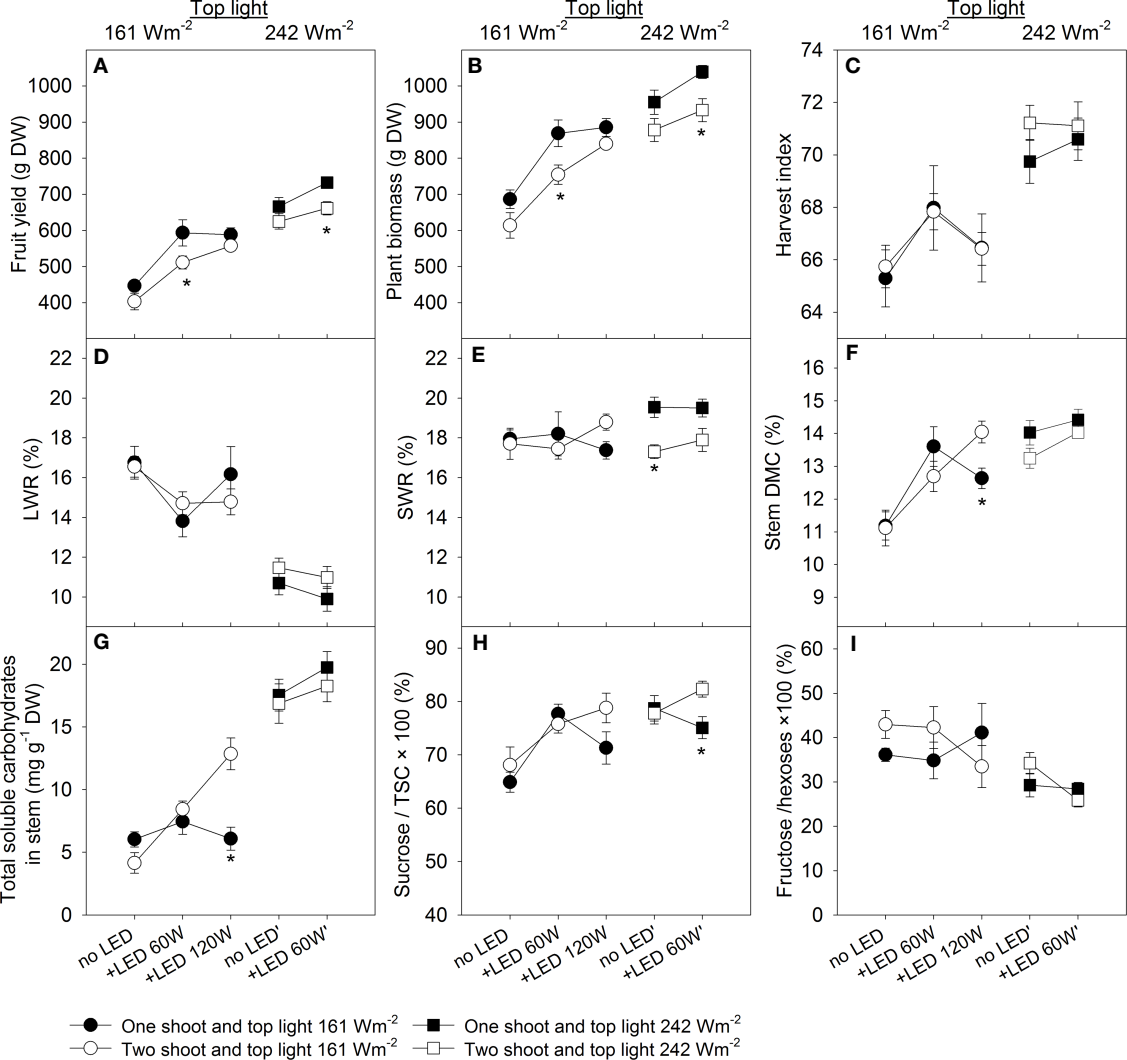
The effects of HPS top light (161 W or 242 W m^-2^) and LED inter-lighting on tomato yield, biomass, and other physiological traits. In the compartment with low top light supplemental LED inter-lighting was added at the levels 0, 60, and 120 W m^-2^ and in the compartment with high top light, the LED inter-lighting was added at the levels 0 and 60 W m^-2^. Each treatment is represented with 5 replications with 2 shoots. **(A)** Tomato fruit yield in g DW per shoot. **(B)** Plant biomass in g DW per shoot (leaves, stem, and total fruit yield). **(C)** Harvest index, defined as total DW fruit yield/total DW plant biomass × 100%. **(D)** Leaf weight ratio, leaf dry matter/total plant biomass × 100% (LWR (%)). **(E)** Stem weight ratio, stem dry matter/total plant biomass × 100 (SWR (%)). **(F)** Stem dry matter content, DW of stem (g DW)/fresh weight (DW) of stem × 100 (DMC (%)). **(G)** Total soluble carbohydrates (TSC) (mg g^-1^ DW) in the stem (n=4-12). **(H)** Sucrose/total soluble carbohydrates (%) in the stem (n=4-12). **(I)** Fructose/hexoses × 100 (%) in the stem (n=4-12). The data were presented as mean values ± se. The error bars show ± standard error. A star represents a statistically significant difference between one- and two-shoot plants within one light treatment (LSD test significant difference comparison, p < 0.05).

We carried out a three-factorial ANOVA to estimate the effects and interactions between branching (one- or two-shoot plants), level of top light intensity, and the use of supplemental LED inter-lighting. All factors significantly affected yield. Under low top light, 60 W m^-2^ LED inter-lighting led to yield increases of 32.8% for one-shoot and 26.8% for two-shoot plants. However, under high top light, the increases were more modest at 10.0% and 5.9%, respectively. For one-shoot plants, increasing LED inter-lighting to 120 W m^-2^ resulted in a yield increase of 31.6%, which was almost the same as the increase observed with 60 W m^-2^, suggesting to advantage in further increasing inter-lighting. In contrast, two-shoot plants showed a yield increase of 38.3% with the highest LED inter-lighting (120 W m^-2^) combined with low top light compared to the control (**Figure 2A**).

### Biomass and dry matter allocation

The effects of light treatments and branching on total plant biomass were similar to their effects on fruit yield, indicating that total photosynthesis during plant growth is the main determinant of plant yield (**Figure 2B**). High top light or 60 W m^-2^ inter-lighting at low top light led to more efficient dry matter allocation to generative organs, boosting yield. However, the highest LED inter-lighting (120 W m^-2^) tended to decrease the dry matter allocation to the fruits, indicating that an excessive amount of inter-lighting may negatively affect harvest index (HI) in comparison with the high top lighting (compare 161 W + 120 W vs. 242 W + 60 W m^-2^). No difference was found in dry-matter allocation to generative organs between one- and two-shoot plants (**Figure 2C**; **Table S1**).

Likewise, no significant difference was found between one- and two-shoot plants for dry matter allocation to leaves (LWR) (**Figure 2D**; **Table S1**). Both high top light and supplemental LED inter-lighting (60 W m^-2^) reduced LWR. Interestingly, the highest LED inter-lighting level (120 W m^-2^) tended to increase rather than decrease dry matter allocation to leaves (**Figure 2D**). This might be because carbohydrate utilization from leaves was limited due to a more uniform light distribution along the canopy. Dry matter allocation to the stems remained unchanged across light treatment (**Figure 2E**; **Table S1**). Under high top light intensity, two-shoot plants allocated less dry matter to the stem than one-shoot plants. This difference is attributable to the two-shoot plants’ structure, where a single stem from roots to the first inflorescence connects both shoots. However, extremely high LED-inter-lighting (120 W m^-2^) tended to increase dry matter allocation to the stems of two-shoot plants, indicating better DM allocation from leaves in two-shoot plants than in one-shoot plants under this condition.

### Stem dry matter content

Dry matter content (DMC, %) in the stem can indicate the accumulation of non-structural carbohydrates. Both top light and 60 W m^-2^ LED inter-lighting increased stem DMC, with a more noticeable effect under lower top light (**Figure 2F**). No significant difference in stem DMC was observed between one- and two-shoot plants. However, their responses to the highest LED inter-lighting level varied: Stem DMC decreased for one-shoot plants at 120 W m^-2^ compared to 60 W m^-2^, while it increased for two-shoot plants. The elevated stem DMC in two-shoot plants (**Figure 2F**) aligns with their higher dry matter allocation to the stems under the highest LED inter-lighting level (120 W m^-2^) (**Figure 2E**).

### Stem soluble carbohydrates

Consistent with the observed effects on DMC, both top and inter-mediate lighting enhanced the levels of total soluble carbohydrates (**Figure 2G**). The interaction among top lighting, inter-lighting, and branching was significant for the Sucrose/TSC value (**Figure 2H**; **Table S1**), indicating that branching responses vary based on lighting combination. Specifically, our data showed that both plant types exhibited an increased Sucrose/TSC ratio in the stem with inter-lighting under low top light. However, their responses diverged under high top light: One-shoot plants tended to exhibit a decrease in Sucrose/TSC value, while two-shoot plants tended to exhibit an increase. The ratio of fructose to total hexoses was predominantly influenced by top lighting, with a decrease observed at the high lighting level (**Figure 2I**).

### Leaf physiological traits in relation to canopy position

**Figure 3:**
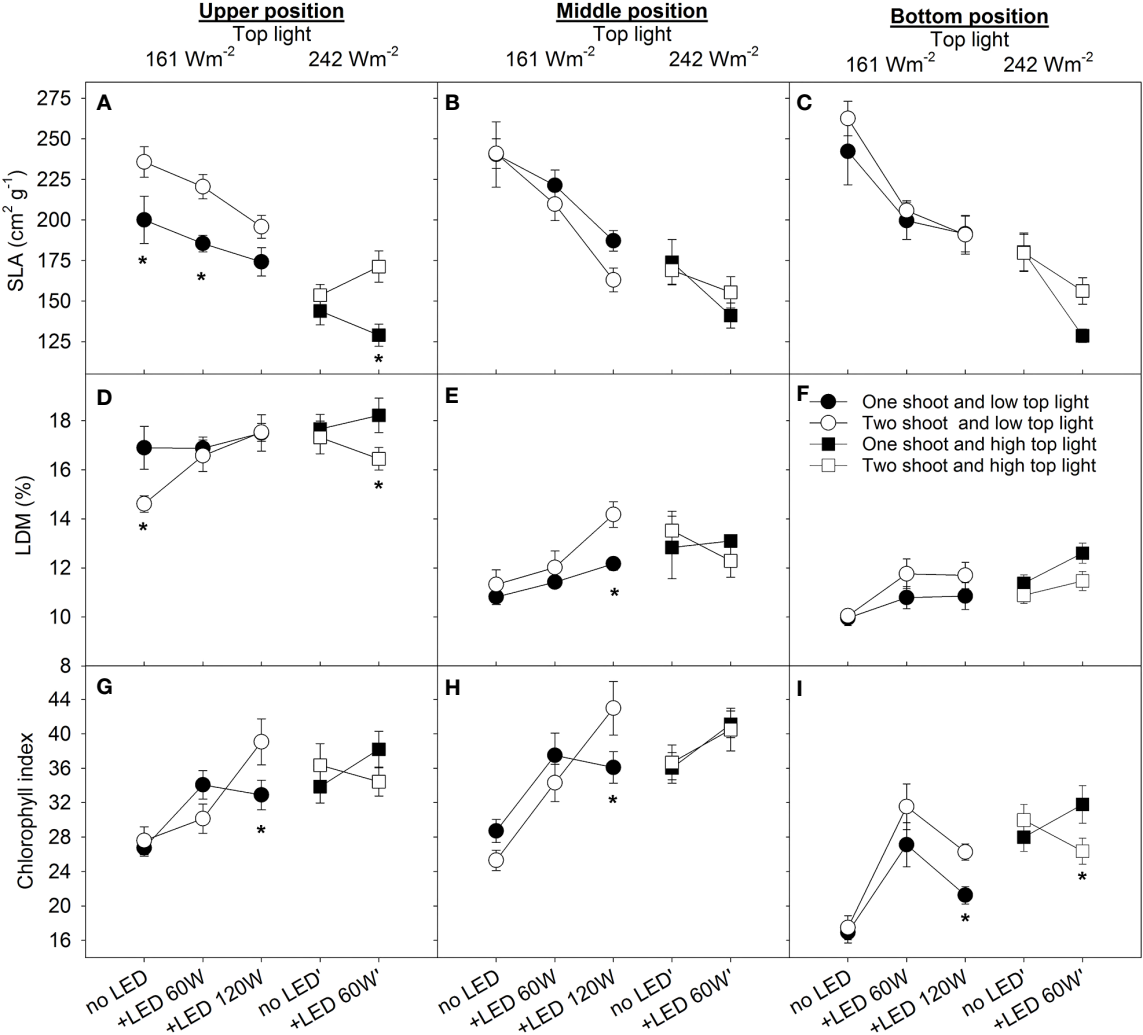
The effect of top lighting, the supplemental LED inter-lighting, and shoot branching (one- or two-shoot plants) on specific leaf area, leaf dry matter content, and chlorophyll index at 3 different 2leaf positions in a tomato canopy.The light conditions were top light at 161 W m^-2^ and 242 W m^-2^ combined with 2–3 levels of supplemental LED inter-lighting (without, 60 W m^-2^, and 120 W m^-2^). The investigated leaves were growing at three different levels of height being representative for 3 different stages of leaf development and different light conditions. The positions were upper (7 developed leaves from apex), middle (height at the level of the lower LED lamp) and bottom (oldest existing leaf) of the plant. Specific leaf area (SLA) (cm^2^ g^−1^), n=10 for each position **(A–C)**, Leaf dry matter (LDM) content in percentage, n=10 for each position **(D–F)**, Chlorophyll index (CL-01 units, n=20 for each position **(G–I)**, bars represent SE. The star represents a statistically significant difference at p<0.05 between one- and two-shoot plants within one light-treatment and calculated with the LSD test.

To assess the differences in source activity among treatments, we estimated the related physiological traits of leaves, such as specific leaf area (SLA), leaf dry matter content (LDM), and chlorophyll index. As the plants were cultivated in a high-wire system, the leaves were analyzed separately at three different positions along the canopy (upper, middle, and bottom). The SLA reflects a strategy of resource allocation within an individual leaf and is negatively related to the leaf thickness and dry matter percentage in leaves. Two-shoot plants showed higher SLA in the top leaves compared to one-shoot plants for almost all treatments (**Figure 3A**). However, no significant differences were observed for the middle and bottom leaves between plant types (**Figures 3B, C**). The treatment with the lowest light intensity (only 161 W m^-2^ top light) generally accounted for the highest SLA, which was associated with low leaf dry matter (LDM) (**Figure 3D**); thus, the higher SLA was not due to thinner leaves but instead to low LDM. The higher SLA for two- than for one-shoot plants under maximum light intensity (242 W top + 60 W m^-2^, **Figure 3A**) can also be explained by a lower LDM (**Figure 3D**).

### Impact of lighting and branching on SLA and LDM

The significant triple interaction was found between top lighting, inter-lighting and branching in relation to SLA and LDM in leaves (**Table S2**). Under low top lighting, inter-lighting similarly reduced SLA for both plant types, a response that can be attributed to both the increased light intensity and the modified light spectrum introduced by the supplemental LED. However, under high top lighting, inter-lighting had a stronger effect on decreasing SLA in one-shoot plants compared to two-shoot plants.

While differences in SLA were noted between the two plant types under conditions of low top light and LED inter-lighting (60 W vs. 120 W m^-2^), LDM remained consistent. This suggests that two-shoot plant leaves were thinner. As expected, the increased light intensity (applied as top or inter-lighting) decreased the SLA. The supplemental LED inter-lighting (60 W m^-2^) reduced SLA more strongly in the bottom leaves than in the top and middle leaves, which may reflect the longer exposure of these leaves to direct supplemental LED lighting.

The middle and the bottom leaves had similar or higher dry matter content (LDM, %) in two-shoot plants than in one-shoot plants (**Figures 3E, F**), indicating that source activity of these two-shoot plants was not the limiting factor leading to reduced plant growth. However, under the 120 W m^-2^ LED inter-lighting condition, a pronounced accumulation of dry matter in middle leaves suggests an imbalance in two-shoot plants: their sink activity was lower than source capacity, leading to increased dry matter storage in these leaves (**Figure 3E**).

### Chlorophyll index responses to lighting and plant type

Both elevated top lighting intensity and supplemental LED inter-lighting increased the chlorophyll index in the leaves. No statistically significant differences were observed between one- and two-shoot plants (**Figures 3G–I**; **Table S2**), suggesting that two-shoot cultivation does not directly affect plant source activity. However, under extremely high inter-lighting, two-shoot plants displayed a higher chlorophyll index compared to one-shoot plants. This suggests that two-shoot plants might be better equipped to adjust to a more uniform light distribution along the canopy.

### Stem elongation response to plant type and lighting

**Figure 4:**
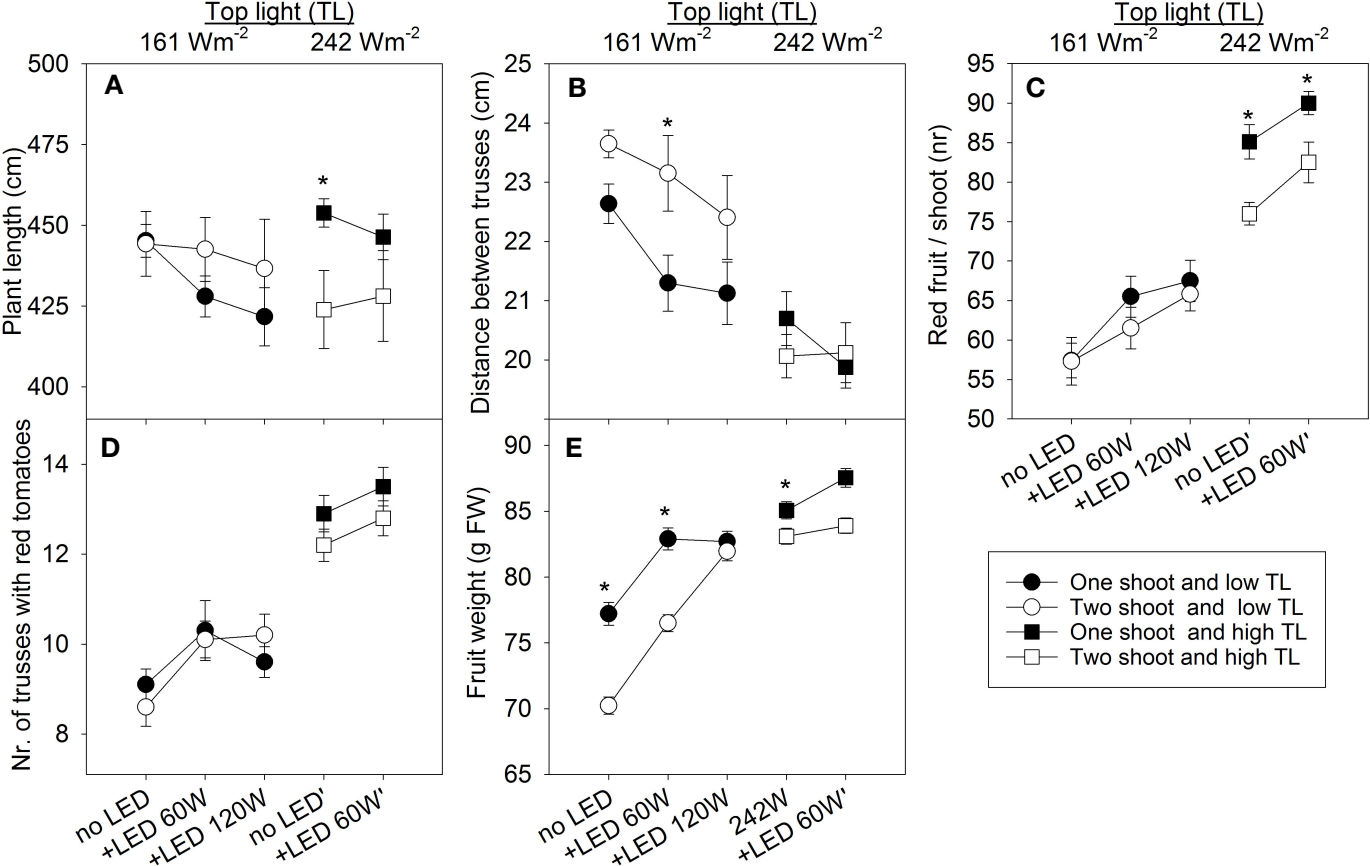
The effect of top light intensities (161 W m^-2^ and 242 W m^-2^) and supplemental LED inter-lighting on plant growth and yield components of greenhouse tomato plants grown as one- and two-shoot plants. The top light intensities were combined with 2 or 3 levels of supplemental LED inter-lighting (no LED, 60 W m^-2^, or LED 60 W m^-2^ with low 120 W m^-2^ top light). **(A)** Plant length (cm) at final harvest (n=10). **(B)** Distance between trusses (plant length/total number of fruit trusses) (n=10 for each treatment). **(C)** Number of red fruit/shoot (n=10/treatment). **(D)** Sum of all fruit trusses that carried at least one red tomato during the entire growth period. **(E)** Average fruit weight (g FW) of all red tomatoes (n=573–900). The data are presented as mean values ± SE. The star represents a statistically significant difference at p < 0.05 between one- and two-shoot plants within one light-treatment and calculated with the LSD test.

Under low top lighting, two-shoot plants typically exhibited greater length than one-shoot plants. In contrast, with high top lighting, two-shoot plants were, or tended to be, shorter (**Figure 4A**). A significant portion of these length differences can be attributed to the distance between trusses (**Figure 4B**), indicating that stem elongation is a primary factor contributing to these differences.

### Fruit count and truss formation

Tomato plant yield is a product of both the number of fruits and the individual weight of each fruit. Our findings indicate that the number of fruits increased with higher top light, supplemental 60 W m^-2^ LED inter-lighting, and during one-shoot plant cultivation (**Figure 4C**). Generally, the fruit count corresponds to the truss count per shoot (**Figure 4D**), as all tomato trusses were standardized to seven fruits per truss each during their formative phase. The reduced number of trusses in two-shoot plants can be explained by their structure, where the second shoot is preserved in the leaf axis just below the first flower truss. This structure delays truss formation on the second shoot. However, when comparing the main stems, truss numbers remained consistent between one-shoot and two-shoot plants (**Table S3**). The differences in number of trusses bearing at least one ripe tomato were also maintained across the two shoots of two-shoot plants (**Table S4**). The increase of number of trusses with at least one ripe fruit under 120 W m^-2^ inter-lighting appears related to faster ripening, as the total count remained consistent.

### Plant type and lighting affect fruit weight

High top lighting reduced the weight differences between fruits of one- and two-shoot plants. The fruit weight of two-shoot plants was 2.3% and 4.2% less than the fruit weight of one-shoot plants under 0 and 60 W m^-2^ LED, respectively. Under low top lighting, this difference was 9.0% and 7.7% for 0 and 60 W m^-2^ LED inter-lighting, respectively (**Figure 4E**). While 120 W m^-2^ inter-lighting did not further increase one-shoot plant fruit weight, it did enhance the weight of two-shoot plant fruits.

### Fruit weight distribution along the truss

**Figure 5:**
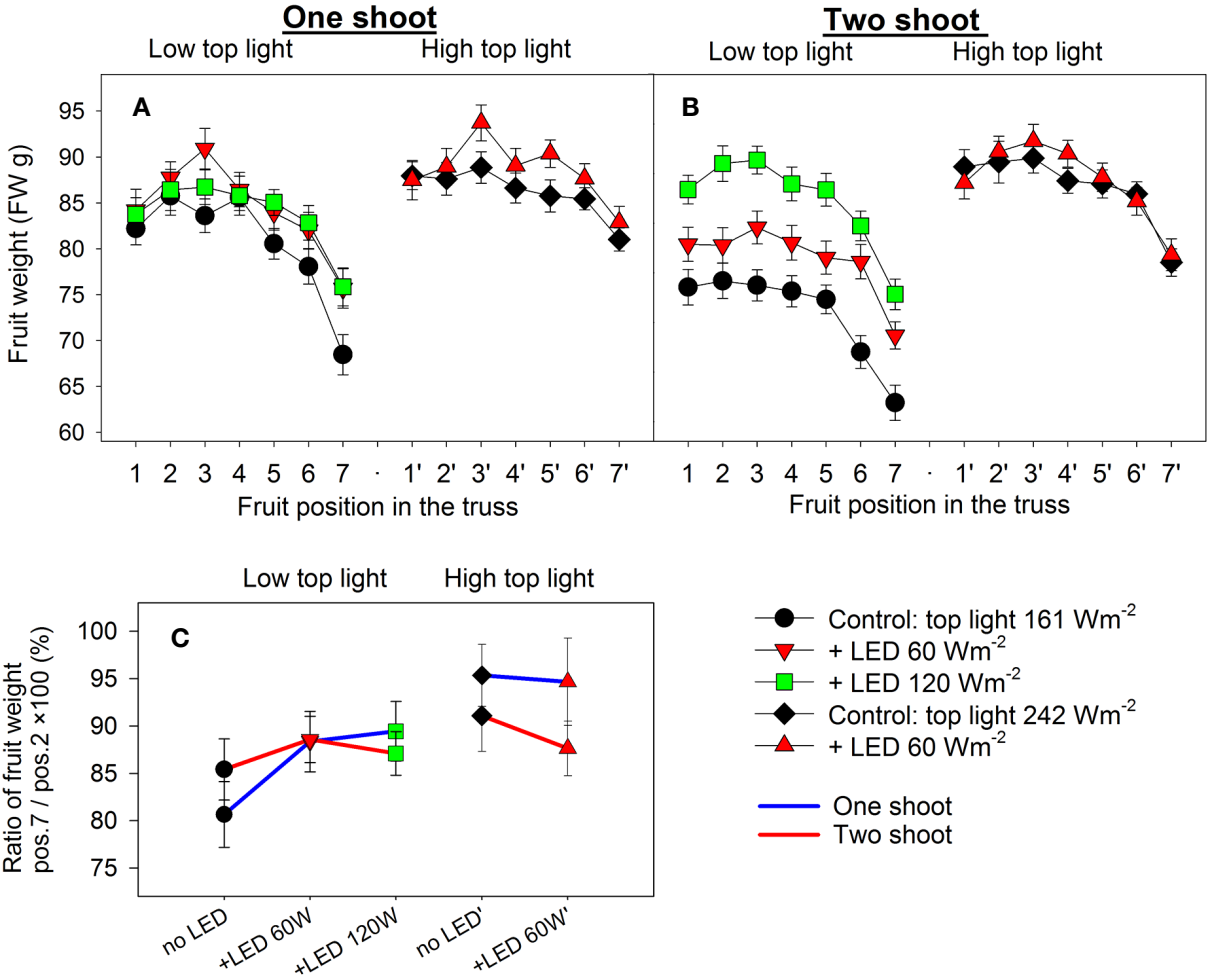
The effect of top lighting, supplemental LED inter-lighting, shoot branching, and fruit position in the truss on the mean fresh fruit weight of a greenhouse tomato. The investigated factors were light source top light at two levels (161 W m^-2^ and 242 W m^-2^) combined with 2 or 3 levels of supplemental LED inter-lighting (no LED, +60 W m^-2^ and +120 W m^-2^ with 161 W m^-2^ top light), shoot branching at 2 levels for one-shoot **(A)** and two-shoot **(B)** plants and fruit position (1–7) in the truss. The mean shown for every treatment is based on 56–119 measurements **(A, B)**. Fruit weight distribution in the truss is represented by the ratio of the mean weight of fruits at position 7 divided by the mean fruit weight at position 2 × 100 (%) **(C)**. Every mean shown is based on the calculated ratio of the averaged fruit weight values (n=10) for 13 trusses. Letters indicate significant differences at p < 0.05. Analysis of the source of variation (3-way ANOVA for factors top light, LED, and shoot) showed significant differences for the factor top light (p<0.013). Analysis of the source of variation (2-way ANOVA for factor LED and shoot) for low top light treatments showed no significant differences. Bars represent standard error (SE). Trusses were pruned to have 7 tomato fruits; trusses with fewer than 7 tomatoes were excluded from analysis.

Analysis of fruit weight at positions 1—7 in the truss showed that two-shoot cultivation led to reductions in both proximal and distal fruit weights under low top light conditions (**Figures 5A, B**). However, this trend was not observed under the highest LED inter-lighting (120 W m^-2^). Despite weight reduction, the ratio between the distal and proximal fruit weights remained consistent in both one- and two-shoot plants (**Figure 5C**). This consistency suggest there was no heightened competition among fruits for assimilates. Consequently, the decline in fruit weight in two-shoot plants was not due to altered source activity. Conversely, both top light and LED inter-lighting under low top light increased or tended to increase the ratio between distal to proximal fruit weights, supporting our suggestion that increased source activity due to increased light intensity mitigated the competition between apical and proximal fruits.

### Diurnal growth rate of fruits: one-shoot vs. two-shoot plants

**Figure 6:**
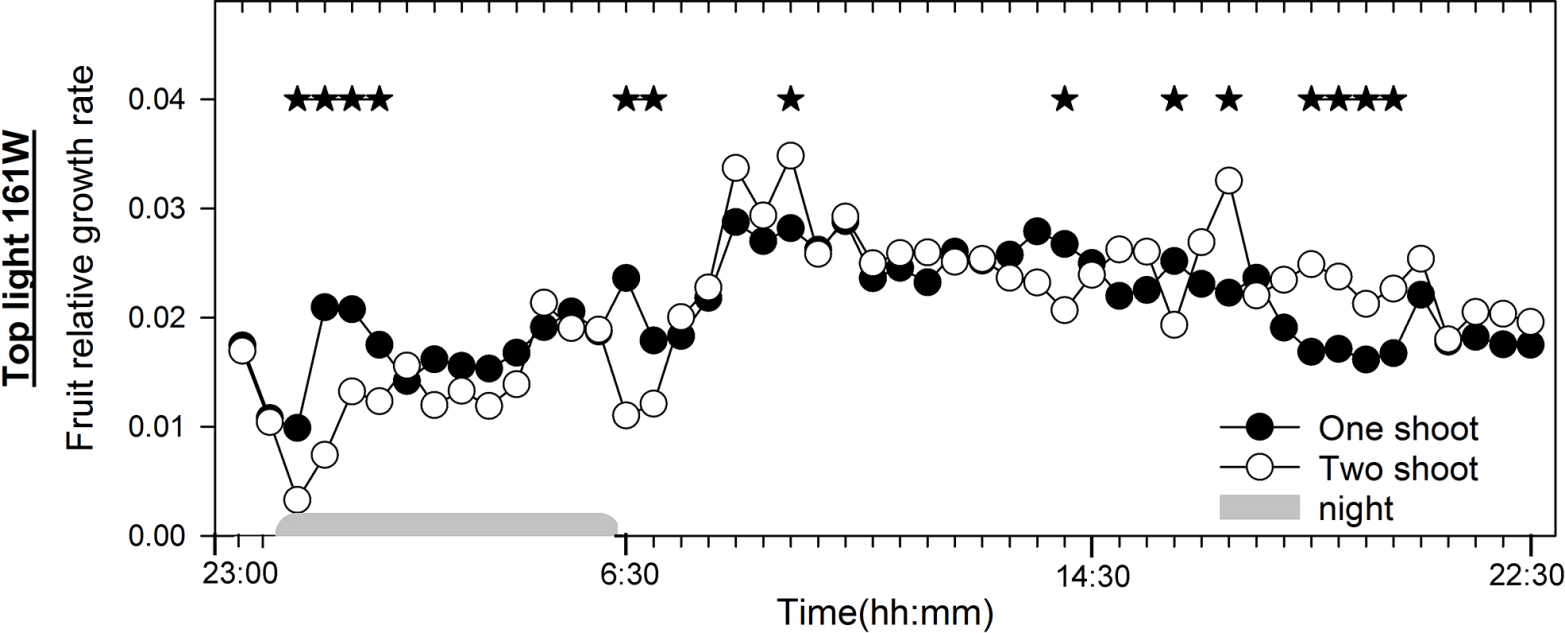
Diurnal fruit growth in one-shoot and two-shoot tomato plants. Relative changes in fruit diameter during the day for one- and two-shoot plants receiving 161 W m^-2^ top light. Every mean is based on 15 and 13 measurement for one- and two-shoot plants, respectively. The experiment was analyzed using two-factor analysis of variance (ANOVA). The first factor was shoot (S) and the second factor was time (T) (30 min intervals). Analysis of the source of variation (ANOVA) showed a significant interaction between S and T at p < 0.001. Stars indicate significant differences at p < 0.05. The night period was from 00:15 to 06:15.

To better understand the physiological factors affecting fruit size, we analyzed the diurnal growth rate of fruits of one- and two-shoot plants under conditions of low top light without LED, where the most significant differences in average fruit size were observed due to shoot branching (**Figures 5A, B**). We found that two-shoot cultivation decreased the relative fruit growth shortly after a change in the light conditions: during the first 2 h of the dark period and the first hour of the light period. This decrease in relative fruit growth rate was largely offset by accelerated growth in the latter half of the light period (**Figure 6**).

### Fruit quality assessment

**Figure 7:**
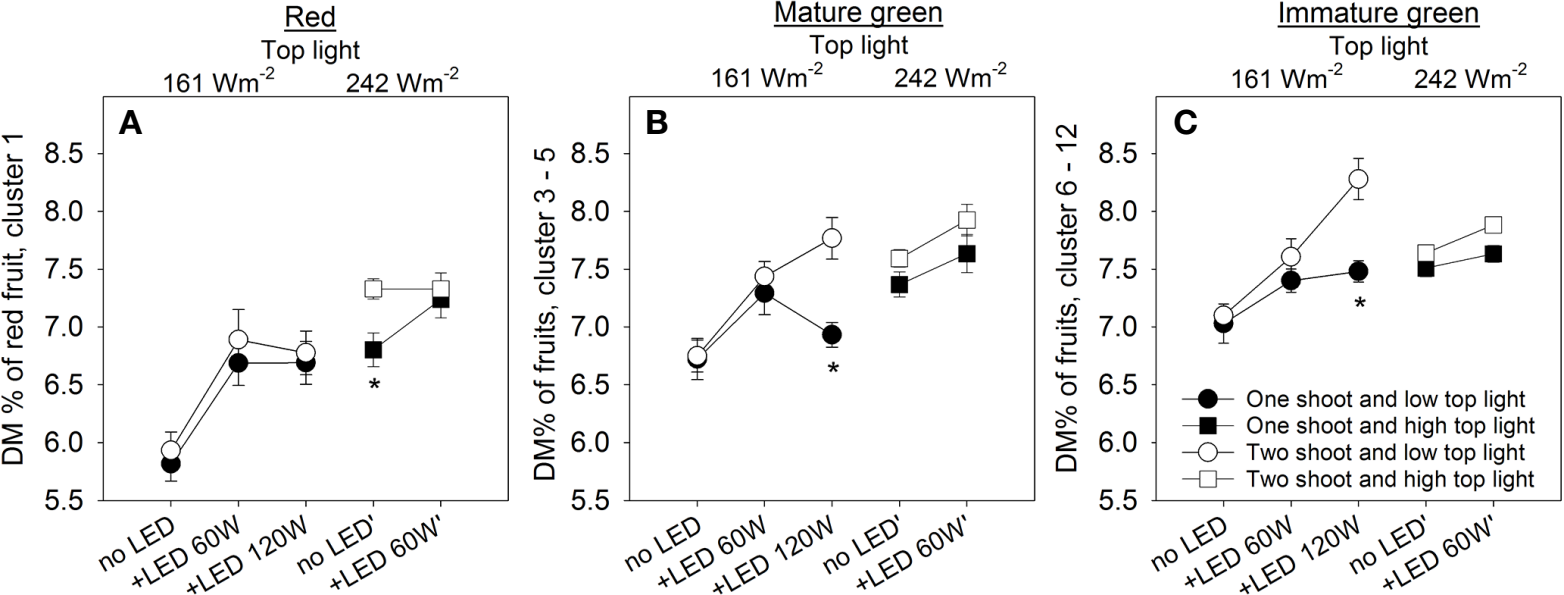
The effect of top light intensities, supplemental LED inter-lighting, shoot branching, and fruit development stage on dry matter percentage (DM%) of tomato fruits. The investigated factors were light source top light at two levels (161 W m^-2^ and 242 W m^-2^) combined with 2 or 3 levels of supplemental LED inter-lighting (no LED, +60 W m^-2^, and +120 W m^-2^ with 161 W m^-2^ top light), shoot branching at 2 levels (one- and two-shoot plants) and 3 fruit developmental stages. Mean dry matter percentage of red mature fruits for lowest truss (n=10) **(A)**. Mean dry matter percentage (DM%) of green tomatoes from trusses 3–5 (n=30) at the height of the lower LED lamp **(B)**. Mean dry matter percentage of all green tomatoes from trusses 6–12 (n=10), which were above the lowest LED lamp **(C)**. The data are presented as mean values ± SE. Stars indicate significant differences at p < 0.05 (LSD test) for one- and two-shoot plants within the same light treatment.

**Figure 8:**
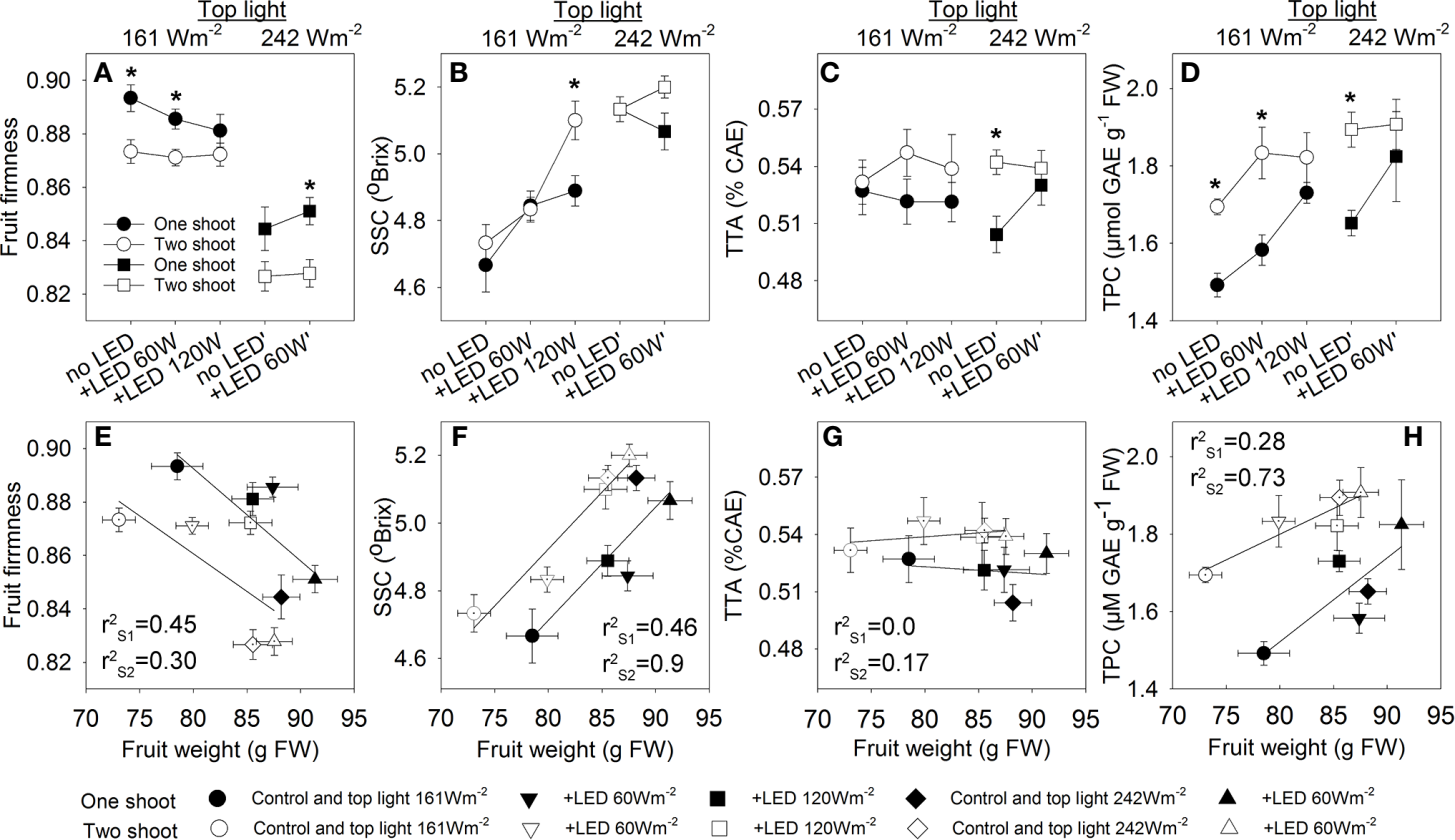
The effect of top light intensities, supplemental LED inter-lighting, and shoot branching on tomato fruit quality. The investigated factors were top lighting at two levels (161 W m^-2^ and 242 W m^-2^) combined with 3 or 2 levels of supplemental LED inter-lighting (no LED, +60 W m^-2^, and +120 W m^-2^ with 161 W m^-2^ top light) and shoot branching at 2 levels (one-shoot [black] and two-shoot [white] plants). For the quality analysis, tomato fruits at position 3 in a truss were collected on 3 harvesting days during the growth period. The data are presented as mean values ± SE. The stars represent statistically significant differences at p < 0.05 between one- and two-shoot plants within the same light treatment (based on the LSD test) **(A–D)**. Relationships between tomato weight and tomato fruit quality traits presented. The r^2^ values of the regression line were calculated for all 5 treatments of the one-shoot (SL) and two-shoot (DL) plants **(E–H)**. Fruit firmness was based on a scale from 0 to 1, where “0” means lack of firmness and “1” is full firmness, determined with a Durofel firmness tester (n=9) **(A, E)**. Soluble solid content (SSC) (expressed as °Brix) was measured with a digital refractometer (PR-101α 293; ATAGO, Japan) (n=9) **(B, F)**. Total titratable acidity (TTA) was determined using an automatic titrator (794 Basic Titrino 294; Metrohm, Switzerland) and expressed as percentage of citric acid equivalents (CAE) g^-1^ FW. (n=6) **(C, G)**. Total phenolic compounds (TPC) in tomato fruits (mg GAE 100 g ^−1^ FW; n=3-6) **(D, H)**.

Assessment of quality is made via a set of recognized parameters, and fruit dry matter (DM) is a valuable indicator of quality that is linked to many aspects of fruit cultivation. Two-shoot plants tend to have a higher DM% in fruits at all fruit development stages (**Figures 7A–C**). As expected, both top light and LED inter-light increased the DM% in the fruits, supporting the positive contribution of source activity to dry matter accumulation in the fruits. Other crucial attributes determining fruit quality include firmness, soluble solids content (SSC), total titratable acidity (TTA), and total phenolic content (TPC). Two-shoot plant cultivation affected not only the fruit size and DM% of fruits but also the quality traits. Specifically, the fruits of two-shoot plants showed decreased fruit firmness. Cultivation under high top light also decreased fruit firmness, whereas supplemental LED inter-lighting had no significant effect (**Figure 8**; **Table S6**). Two-shoot plant cultivation did not change fruit SSC, while high top light strongly increased SSC. Extremely high LED-inter-lighting increased SSC in fruits of two-shoot plants but had only a weak effect on one-shoot plants (**Figure 8B**).

Two-shoot plants’ fruit increased total acidity (TAA) irrespective of light treatments (**Figure 8C**). Phenolic accumulation varied with shoot branching and light intensity. Under low-top light, two-shoot plants showed significantly enhanced accumulation of phenolic compounds in fruits; however, this effect was absent at extremely high inter-lighting (120 W m^-2^) or with the combination of high-top light and supplemental LED inter-lighting.

The phenolic concentration in the fruits was positively correlated with DM%, SSC, and TTA (**Table S7**), indicating that the higher accumulation of phenolics was not due to increased competition between primary and secondary metabolism but due to excess C-skeletons that were used in both primary and secondary metabolic activities. The correlation between fruit weight and fruit quality traits revealed that the responses of one- and two-shoot plants to varying light conditions were similar; however, the sensitivity was different. At the same fruit weight, two-shoot fruits had lower firmness, but higher SSC, TTA, and total phenolic content (**Figures 8E–H**).

### Plant hormone composition in xylem sap

**Figure 9:**
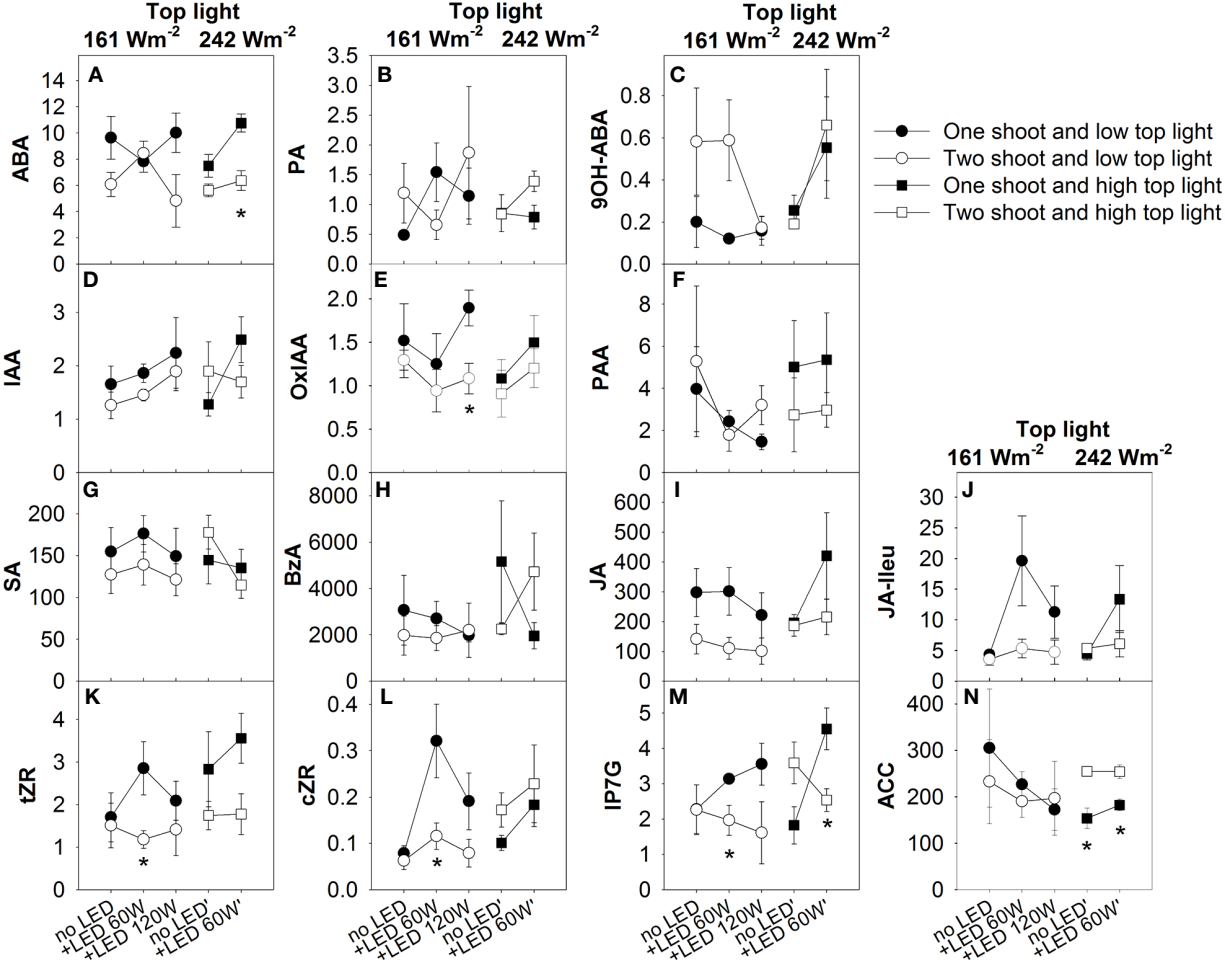
The effect of top light intensities, supplemental LED inter-lighting distribution, and shoot branching on the plant hormone composition of the xylem sap at final harvest. The studied factors were top lighting at two levels (161 W m^-2^ and 242 W m^-2^) combined with 3 or 2 levels of supplemental LED inter-lighting (no LED, +60 W m^-2^, and +120 W m^-2^ with 161 W m^-2^ top light), and 2 levels of shoot branching (one- and two-shoot plants). Variables are plant hormone concentration in xylem sap (pmol/mL): abscisic acid (ABA) **(A)**, phaseic acid (PA) **(B)**, 9-hydroxy-ABA (9OH-ABA) **(C)** indole-3-acetic acid (IAA) **(D)**,oxo-IAA (OxIAA) **(E)**, phenylacetic acid (PAA) **(F)**, salicylic acid (SA) **(G)**, benzoic acid (BzA) **(H)**, jasmonic acid (JA) **(I)**, and JA-isoleucine (JA-Ileu) **(J)** (n=4–5), trans-zeatin riboside (tZR) **(K)**, cis-zeatin-riboside (cZR) **(L)**, isopentenyl adenine-7-glucoside (iP7G) **(M)**, 1-aminocyclopropane-1-carboxylic acid (ACC) **(N)**. The data are presented as mean values ± SE. The stars represent statistically significant differences at p < 0.05 between one- and two-shoot plants within the same light treatment (LSD test) (n = 5).

To address the question of whether the modulation of fruit size and quality composition of the fruits occurred through the modification of a root-driven signal induced by one of two shoot cultivation, we analyzed the plant hormone composition in the xylem sap (**Figure 9**). Two-shoot plants managed to double the xylem sap flow rate for each root, leading an equivalent xylem sap rate when computed per shoot (**Figure S1**; **Table S9**). A significant reduction in concentration was found for ABA, jasmonate, and cytokinins. Evidently, the hormone flow in the xylem can influence plant development, leaf activity, and fruit sink capacity. Notably, the diminished concentrations of several cytokinins in xylem sap of decapitated two-shoot plants hint at a role for these cytokinins in the observed reduction in sink capacity in fruits of two-shoot plants. The effect of top light on fruit development seems to act independently of the regulation of hormone composition in xylem sap, as top light intensity did not affect hormone concentrations. On the other hand, LED-inter-lighting was able to increase JA-Ileu and cZR, though the increase in JA-Ileu was only noticeable in one-shoot plants.

A significant three-way interaction among top lighting, inter-lighting, and plant branching (one- or two-shoot plants) on ABA and PA concentration was determined by the varying responses of ABA and PA accumulation in xylem sap (**Figure 9**; **Table S8**). Under low top lighting conditions, inter-lighting markedly enhanced ABA accumulation in two-shoot plants compared to one-shoot plants. However, when top lighting was high, the inter-lighting effect on ABA accumulation reversed, favoring one-shoot plants. In contrast, PA concentration exhibited an opposing response: at low top lighting, inter-lighting increased PA concentration in one-shoot plants, while at high top lighting, a positive inter-lighting effect was only observed for two-shoot plants.

## Discussion

The primary challenge faced by tomato growers is that achieving high yields in greenhouse cultivation often coincides with reduced fruit quality, characterized by lower sugar content and altered phytochemical composition. Therefore, an understanding of the mechanisms guiding both fruit size and quality is crucial for the optimization of tomato production in greenhouses, as this knowledge will identify practices that allow both high yield and high quality of greenhouse tomatoes. Factors known to affect fruit weight, yield, and quality include top light intensities, LED inter-lighting, and shoot branching in one- and two-shoot plants, leading to the question of whether synergetic or antagonistic interactions occur between these factors.

Our study reveals distinct effects of lighting and two-shoot branching on fruit size and other quality traits. Specifically, enhanced lighting increased fruit size, while two-shoot cultivation decreased it. However, both enhanced lighting and two-shoot cultivation positively influenced fruit quality traits. The present study did not identify a significant interaction between light conditions and shoot branching concerning fruit size and fruit quality traits (**Tables S5**, **S6**). This suggests that these treatments affect different physiological processes that guide fruit development. While light primarily boosts source activity by directly increasing leaf photosynthesis per leaf area, the effects of two-shoot cultivation on plant growth and development remain underexplored. Our findings indicate that two-shoot cultivation reduces fruit size and modulates fruit quality, primarily through a decrease in the sink capacity of the fruits, rather than by any effect on source activity.

### Two-shoot plants reduce the sink capacity of fruits

The evidence that source activity did not limit fruit size was based on the comparison of the major leaf-related traits that characterize plant source activity. Specifically, the dry matter allocation to the leaves, leaf chlorophyll content, SLA, and accumulation of dry matter in the leaves were all found to be the same or higher in two-shoot plants when compared to one-shoot plants. Furthermore, analysis of soluble sugars in the stem indicated that two-shoot plants accumulated similar amounts of soluble sugars, which can be used as carbohydrate resources for fruit growth, compared to one-shoot plants. These findings further support the suggestion that the modulation of source activity was not responsible for the changes in fruit size observed in two-shoot plant cultivation.

Another indication that sink capacity, rather than source activity, was the main determinant of fruit size came indirectly from the comparison of the individual fruit weights located at proximal and distal positions in the truss. Usually, larger fruits develop at the proximal positions (closer to the stem and the first develop) than at the distal position (Bangerth and Ho, 1984). The consistent distal/proximal fruit weight ratios between one- and two-shoot plants suggest that two-shoot cultivation does not alter fruit competition during development. However, both top light and inter-lighting decreased the relative difference between distal and proximal fruits (**Figure 5**), supporting the assumption that this trait is sensitive to the amount of available carbohydrates, and thus to source activity. A positive effect of inter-lighting in reducing the difference between distal and proximal fruits was also demonstrated in our previous study (Paponov et al., 2020).

More evidence that the sink capacity limits fruit size is based on the observation that the fruits of two-shoot plants at all developmental stages have equal or higher dry matter content. The dry matter content in growing fruits consists of non-structural carbohydrate (Serio et al., 2004); therefore, the dry matter content in fruits can also reflect the balance between source activity and sink capacity in the plants. Under source-limiting conditions (low light intensity), both fruit size and dry matter content in the fruits decreased (**Figures 4E**, **7**). These decreases are in agreement with the fact that cell expansion in the fruits is sensitive to the level of assimilates supplied to the fruits during the fruit loading phase (Bertin and Genard, 2018), whereby the dynamics of dry matter accumulation in the fruits reflect the balance of water and carbohydrate fluxes to the fruits (Guichard et al., 2001). However, the ability of two-shoot plants to generate fruits with higher dry matter content at a specific fruit weight indicates alternative regulation of solute fluxes into the fruits when the fruit size is not limited by the availability of assimilates, but by sink capacity. The higher dry matter accumulation in the fruits of two-shoot plants can reflect a higher contribution of phloem flux than xylem flux for fruit formation (Hanssens et al., 2015).

The capacity of two-shoot plants to build fruits with higher dry matter content at a specific fruit size might be related to the fact that the final fruit size is defined shortly before the beginning of the fruit loading stage, when the ultimate number of cells in a fruit is determined (Bertin et al., 2003). This number of cells can limit the ultimate size of the fruit, even if carbohydrate availability during fruit loading is not limited. Thus, the smaller fruit size of two-shoot plants at maturity might reflect a reduced sink size due to the formation of a smaller number of cells during the cell division stage. Restriction of fruit size due to less cell division before fruit loading, together with maintenance of carbohydrate fluxes to the fruits during the loading phase, will generate a fruit with a smaller size but higher accumulation of dry matter and other modifications of fruit quality traits.

### Potential hormonal regulation of sink capacity in two-shoot plants

In two-shoot plants, plant hormones, particularly cytokinins, appear to play a role in modulating sink capacity. Our data reveals a notable reduction in cytokinin levels in the xylem sap of these plants, especially under conditions of low top light with LED inter-lighting. This reduction might be associated with the diminished sink capacity observed in their fruits. Parallel findings from studies on two- branched beans and peas also reported a decline in xylem sap cytokinin concentration in two-shoot plants (Bangerth, 1994; Li et al., 1995; Kotov and Kotova, 2018). The trend has been attributed to an increased auxin transport to the roots due to the additional shoot, which subsequently suppresses cytokinin synthesis, leading to its reduced presence in the xylem sap.

Hormonal pathways, especially involving cytokinins, are also integral in modulating fruit size and quality under stress conditions. For instance, drought and osmotic stress have been shown to reduce cytokinin levels (Kudoyarova et al., 2006; Gujjar and Supaibulwatana, 2019). Given the central role of cytokinins in regulating fruit cell division (Zhang and Whiting, 2011; Matsuo et al., 2012), a systemic decrease in their levels might impact fruit sink capacity and strength.

However, cytokinins alone can’ t explain all fruit weight variation. For instance, under low top lighting without LED, fruit weight differences persisted despite similar cytokinin concentration in both plant types. This suggests other hormonal influences at play. Observations indicate that two-shoot plants under low top lighting display variations in hormone concentrations particularly a rise in 9OH’-ABA, known for its ABA-like activities (Zhou et al., 2004). The role of this hormone in sink activity warrants further exploration.

### Phloem and xylem flux modulation in two-shoot plants

In addition to the regulation of fruit size through hormone pathways, the intricate balance between xylem and phloem solute transport is crucial in shaping fruit size and quality. One possible mechanism that could contribute to the accumulation of dry matter content in fruits (and the improvement of other fruit quality traits) is the reduction in xylem flux relative to phloem flux directed to fruits. This phenomenon has been observed under environmental stresses like drought and salt stress. Here, heightened nutrient concentrations due to these stresses result in a decreased solute flux from roots, attributed to reduced root pressure (Ehret and Ho, 1986; Araki et al., 2001; Van de Wal et al., 2017; Cui et al., 2020).

In the context of two-shoot plants, the enhanced fruit quality is not easily attributed to reduced root pressure, as the two-shoot plants were able to duplicate the xylem sap flow rate per root, resulting in the same xylem sap rate calculated per shoot (**Figure S1**; **Table S9**). This maintained pressure cannot be explained by an increased amount of carbohydrates transported to the roots because increased carbohydrate biosynthesis due to increased light intensity did not enhance root exudation rate in either one- or two-shoot plants. Intriguingly, a previous study showed increased xylem sap flow rate with supplemental LED inter-lighting (Paponov et al., 2020). Such discrepancies might arise from the use of different genotypes or varied experimental setups. Moreover, xylem sap flux regulation might exhibit a circadian rhythm, as observed in young maize plants (Lopez et al., 2003).

The nighttime reduction in fruit growth could be indicative of a dip in xylem sap pressure, given that root pressure is the primary solute supply source during this period (Hanssens et al., 2015). The consistent xylem sap rate per shoot (**Figure S1**; **Table S9**) suggests that the transient reduction in fruit growth during the initial 2 h of the night, and shortly after lights are turned on, cannot be explained by a reduced xylem sap flow rate. Instead, the distinct architecture of two-shoot plants might increase resistance to xylem flow, particularly during pivotal water supply periods, such as during light transitions. This restricted solute supply from the xylem to fruits for two-shoot plants might be due to increased hydraulic resistance associated with the reduced xylem area, vessel numbers, and vessel size (Lang and Ryan, 1994; Choat et al., 2009).

Abrupt changes in greenhouse lighting like turning lights on or off, can initiate these trigger critical periods. Turning light on promotes morning water movement via transpiration, with leaves drawing from the root-extended water column. Conversely, turning lights off decreases transpiration, positioning root pressure as the main force pushing the water flux into the plant’s aboveground parts. The observed decline in fruit growth after abrupt light changes suggests that the two-shoot architecture’s adjusted hydraulic resistance might influence the final fruit quality by reducing xylem flux to fruits.

### The interplay of shoot branching and light on fruit quality

Fruit size and metabolite accumulation are largely determined by sink capacity and solute transport via phloem and xylem. Both of these processes can be modulated by plant architecture and light conditions. Understanding their combined effects can guide adjustments for appropriate fruit quality traits. One key aspect for fruit quality is the balance between sugars and organic acids, which plays a pivotal role in determining tomato flavor (Agius et al., 2018). Our observations show that while two-shoot plants maintain a consistent SSC, they exhibit an increase in total acidity (**Figures 8B, C**, **Table S6**). In contrast, increasing light intensity boosts SSC without affecting total acidity, highlighting significance of source activity in the accumulation of soluble compounds in fruits.

Delving deeper into the metabolic dynamics, the elevated organic acid content in fruits from two-shoot plants might reflect the dynamics of sugars and organic acid use for respiration during fruit maturation. As fruit develop, the roles of sugars and organic acids in respiration evolve. Notably, as fruits approach maturity, sugars become predominantly stored in vacuoles, rendering them less accessible for respiration (Coombe, 1976). This dynamic could lead to a shift in respiratory substrates from sugars to organic acids, particularly citrate. Given the smaller fruits size typical of two-shoot plants, it is plausible that these fruits exhibit reduced respiratory activity as they near maturity. This would result in a decreased consumption of organic acids for respiration, leading to their heightened content at full maturity (**Figure 8C**; **Table S6**).

Fruit firmness is another complex quality trait and can be influenced by cellular turgor pressure. A strong positive correlation exists between fruit firmness and the content of dry matter or total soluble solids, as observed in tomatoes (Saha et al., 2009; Aurand et al., 2012) and kiwis (Nardozza et al., 2011). However, the diminished firmness of fruits from two-shoot plants (**Figure 8A**), despite their greater accumulation of dry matter (**Figure 7**), suggests other underlying factors affecting firmness. One such factor could be the number of cells in the fruits. Fruits with smaller cells and more cell structures tend to exhibit higher mechanical resistance. Indeed, comparisons across various tomato genotypes have indicated that fruits with smaller cells and more cell structures are typically firmer (Aurand et al., 2012). While we did not quantify cell numbers in our tomato fruits, our findings underscore that a limited sink capacity was the main factor restricting fruit growth, leading to the assumption that the number of cells was reduced in the fruits of two-shoot plants. The excess carbohydrates available during fruit loading might contribute to a stronger elongation of fruit cells of two-shoot plants, and this would ultimately reduce firmness. Interestingly, plants given high top lighting also showed decreased fruit firmness despite the higher accumulation of dry matter content or SSC in the fruits. This phenomenon could be attributed to elevated temperatures in the high top light compartment, as previous studies have linked high temperature to reduced fruit firmness (Hertog et al., 2004).

Greenhouse-grown tomato plants frequently experience sub-optimal lighting, which can lead to reduced accumulation of both primary and secondary metabolites. Given the health benefits associated with many secondary metabolites, the observed increase in phenolic compounds in two-shoot cultivated tomatoes underscores the potential advantages of this cultivation approach in greenhouses. The interplay between primary and secondary metabolism might favor the accumulation of secondary metabolites at the cost of primary ones (Herms and Mattson, 1992).

The elevated accumulation of phenolic compounds in fruits from two-shoot plants can be partly attributed to their smaller size and the varied distribution of these compounds within the fruit. Tomato phenolic concentrations differ across fruit sections, with the highest levels found in epidermal and placental tissues. For example, an analysis of flavonol distribution in Spanish cherry tomatoes showed that 98% of the total flavonols occurred in the skin (Stewart et al., 2000). This localized accumulation aligns with other research, highlighting that a majority of certain flavonols and quercetin derivatives are predominantly found in the epidermis (Moco et al., 2007; Slimestad and Verheul, 2009). Due to their reduced size, fruits from two-shoot plants have an enhanced surface-to-volume ratio. Given the skin’s rich phenolic compound content, its increased proportion in these smaller fruits likely contributes to a higher overall phenolic content. Additionally, our findings indicate that enhanced light intensity increased both SSC and total phenolics (**Figures 8B, D**), suggesting a redirection of excess carbohydrates towards the synthesis of secondary metabolites.

## Conclusion

The balance between source strength, which comes from plant photosynthesis, and sink capacity, mainly determined by the number and potential size of fruits, is crucial for plant growth and performance (Lemoine et al., 2013). Understanding the relationships between source and sink, especially the transport and partitioning of assimilates to different organs, can help improve fruit yield and quality (Ho, 1996). Our study indicates that greenhouse practices, such as creating two-shoot plants through shoot branching and using supplemental lighting, can independently regulate the size and quality of the produced fruits. Overall, two-shoot branching primarily modified sink capacity, while lighting primarily affected source activity. Two-shoot cultivation reduced the xylem sap concentration of cytokinins that can inhibit the sink capacity of young fruits. Additionally, the increased hydraulic resistance associated with two-shoot plant architecture appears to improve fruit quality due to the higher solute flux from the phloem at the expense of the xylem. The stronger inhibition of sink than source activity, together with the increased hydraulic resistance in the stem, resulted in fruits that were smaller but showed higher accumulation of dry matter content and improved fruit quality traits. Notably, fruits from two-shoot plants had enhanced accumulations of dry matter and phenolic contents. Further investigation of the interactions between shoot branching practices and environmental factors is required to establish the optimal combinations that maximize both yield and fruit quality traits in tomatoes.

## Data availability statement

The original contributions presented in the study are included in the article/**Supplementary Material**. Further inquiries can be directed to the corresponding authors.

## Author contributions

IP, MV and MP designed the experiment. MP performing the majority of the experimental work. MP conducted all data analysis and collaborated with IP on the writing of the paper. PD carried out hormone analysis. All authors provided critical feedback and corrections on the final version of the paper.
