# Supplementary material for: Additive effects of light and branching on fruit size and chemical fruit quality of greenhouse tomatoes
Source: Front Plant Sci. 2023 Oct 24;14:1221163. doi: 10.3389/fpls.2023.1221163 (PMC10628543; doi:10.3389/fpls.2023.1221163)
Supplement: Supplementary file 1 [file DataSheet_1.pdf]

Supplemental data

Table S1. Analysis of variance significance levels. Variables include tomato fruit yield, plant biomass, harvest index (HI), leaf weight ratio (LWR), stem weight ratio (SWR), stem dry matter content (DMC), total soluble carbohydrates (TSC) in the stem, ratio sucrose/TSC in the stem, and ratio fructose/hexoses in the stem. The data was analyzed using a 3-way-ANOVA (A). The first factor was top lighting at two levels (161 W m^{-2} and 242 W m^{-2}) (T). The second factor was supplemental LED inter-lighting at two levels (without LED and 60 W m^{-2}) (L). The third factor was shoot branching at two levels (one- and two-shoot plants). The data were also analyzed using a 2-way-ANOVA (B) for treatments with low top lighting (161 W m^{-2}) only. The first factor was supplemental LED inter-lighting at three levels (no LED, 60 W m^{-2}, and 120 W m^{-2}), and the second factor was shoot branching at two levels (one- and two-shoot plants) (B). Means and SE are presented in Figure 2. Statistically significant effects of factors or interactions between factors are indicated with asterisks: * $p<0.05$, ** $p<0.01$, *** $p<0.001$. The absence of a star denotes no significant difference at a significance level of $p<0.05$.

		**Fruit yield**	**Plant bio-masse**	**HI**	**LWR**	**SWR**	**Stem DMC**	**TSC**	**Sucrose/TSC**	**fruc/hexoses**
A)	**Top light (T)**	***	***	***	***		***	***	***	***
	**LED (L)**	***	***	*	**		***	*	**	
	**Shoot (S)**	***	***			**				
	**T x L**	*	*				*		**	
	**T x S**									
	**L x S**									
	**T x L x S**								*	
B)	**L**	***	***		*		***	**	***	
	**S**	**	**					*		
	**L x S**						*	**		

Table S2. Analysis of variance significance levels. Variables include specific leaf area (SLA), leaf dry matter content (LDM%), and chlorophyll index (Chl) in tomato leaves. The data were analyzed using a 4-way-ANOVA (a). The first factor was top lighting at two levels (161 W m^{-2} and 242 W m^{-2}), the second factor was supplemental LED inter-lighting at two levels (no LED and 60 W m^{-2}), the third factor was shoot branching (one- or two-shoot plants), and the fourth factor was the position of the leaf in the tomato canopy at 3 levels (upper, middle, or bottom).

The data were also analyzed using a 3-way-ANOVA (b) for treatments with low top light (161 W m^{-2}) only. The first factor was supplemental LED inter-lighting at three levels (no LED, 60 W m^{-2}, and 120 W m^{-2}), the second factor was shoot branching at two levels (one- or two-shoot plants), and the third factor was the position of the leaf in the canopy at 3 levels (upper, middle, or bottom).

Means and SE are presented in Figure 3. Statistically significant effects of a factor or interactions between factors was indicated with asterisks, * *p*<0.05, ** *p*<0.01, *** *p*<0.001. NS[1] is close to statistical significance at a probability between 0.05 and 0.064. The absence of a star indicates no significant difference at a significance level of *p*<0.05.

	Top light (T)	LED (L)	Shoot (S)	Position (P)	T x L	T x S	L x S	T x P	L x P	S x P	T x L x S	T x L x P	T x S x P	L x S x P	T x L x S x P
SLA a)	***	***	*	*					**	*	*	NS[1]			
b)		***								**					
DM% a)	***	*		***						*	*				
b)		***		***			*			**					
Chl a)	***	***		***	***										
b)		***		***			**		**	NS[1]					

Table S3. The effect of top light intensities (161 Watt m-2 and 242 Watt m-2) and supplemental LED inter-lighting on number of all trusses individually for every shoot. The data are presented as mean values ± SE. Different letters represent statistically significant differences at $p < 0.05$ between one- and two-shoot plants (for both long and short stems) within each light treatment.

	One-shoot	Two-shoot long	Two-shoot short
Low top	19.7±0.3b	20.0±0.3b	17.6±0.2a
Low top + LED 60W	20.2±0.6a	19.4±0.6a	19±0.8a
Low top + LED 120W	20.0±0.3b	20.4±0.4b	18.6±0.4a
High top	22.0±0.4b	21.8±0.2ab	20.4±0.4a
High top + LED 60W	22.5±0.4b	22.2±0.5ab	20.4±0.9a

Table S4. The effect of top light intensities (161 Watt m-2 and 242 Watt m-2) and supplemental LED inter-lighting on number of trusses that carried at least one red tomato during the entire growth period individually for every shoot. The data are presented as mean values ± SE. Different letters represent statistically significant differences at $p < 0.05$ between one- and two-shoot plants (for both long and short stems) within each light treatment.

	One-shoot	Two-shoot long	Two-shoot short
Low top	9.1±0.3b	9.6±0.2b	7.6±0.5a
Low top + LED 60W	10.3±0.7a	10.6±0.5a	9.6±0.6a
Low top + LED 120W	9.6±0.3a	11.2±0.5b	9.2±0.5a
High top	12.9±0.4a	12.8±0.4a	11.6±0.5a
High top + LED 60W	13.5±0.4a	12.8±0.6ab	12.8±0.6a

Table S5: Analysis of variance significance levels. Variables include length of plants, number of trusses with at least one red tomato per shoot, the distance between trusses, fruit weight, and the number of red mature tomato fruits per shoot. The data were analyzed using a 3-way-ANOVA (A). The first factor was top light at two levels (161 W m^{-2} and 242 W m^{-2}), the second factor was supplemental LED inter-lighting at two levels (without LED and 60 W m^{-2}), and the third factor was shoot branching at two levels (one- and two-shoot plants). The data were also analyzed with a 2-way-ANOVA (B). The first factor was supplemental LED inter-lighting at three levels (without LED, 60 W m^{-2}, and 120 W m^{-2}) and the second factor was shoot branching at two levels (one- and two-shoot plants). Means and SE are presented in Figure 4. Statistically significant effects of factors or interactions between factors are indicated with asterisks: * p<0.05, ** p<0.01, *** p<0.001. The absence of a star denotes no significant difference at a significance level of p<0.05.

		Length of plant	Number of trusses with red tomatoes	Distance between trusses	Fruit weight	Red fruit /shoot
A)	Top light (T)		***	***	***	***
	LED (L)		**	***	***	***
	Shoot (S)				***	**
	T x L					
	T x S	*		*		
	L x S					
	T x L x S					
B)	L		*	***	***	**
	S			*	**	
	L x S					

Table S6: Analysis of variance significance levels. Variables include dry matter (DM)% in tomato fruits at three different developmental stages (Figure 7), tomato fruit firmness, soluble solid content (SSC), total titratable acidity (TTA), and total phenolics (Figure 8). For fruit quality trait analysis, the red mature tomato fruits at position 3 in a truss were harvested (but at all positions for DM% of clusters 6–12). The data were analyzed using a 3-way-ANOVA (A). The first factor was top lighting at two levels (161 W m^{-2} and 242 W m^{-2}), the second factor was supplemental LED inter-lighting at two levels (no LED and +60 W m^{-2}), and the third factor was shoot branching at two levels (one- or two-shoot plants). The data were also analyzed with a 2-way-ANOVA (B). The first factor was supplemental LED inter-lighting at three levels (without LED, 60 W m^{-2}, and 120 W m^{-2}) and the second factor was shoot branching at two levels (one- and two-shoot plants) (B). Means and SE are presented in Figure 7 and Figure 8. Statistically significant effects of factors or interactions between factors are indicated with asterisks: * p<0.05, ** p<0.01, *** p<0.001. NS[1] represents near statistical significance, with a probability of 0.054. The absence of a star denotes no significant difference at a significance level of p<0.05.

		**DM % of red fruits in cluster 1**	**DM % of fruits in clusters 3–5**	**DM % of fruits in clusters 6–12**	**Firmness**	**SSC**	**TTA**	**Total phenolics**
A)	**Top light (T)**	***	***	***	***	***		***
	**LED (L)**	***	***	***				*
	**Shoot (S)**	NS[1]		*	***		*	***
	**T x L**	**						
	**T x S**							
	**L x S**							
	**T x L x S**							
B)	**L**	***	***	***		***		**
	**S**		*	**	***		*	***
	**L x S**		*	*				

Table S7: Correlation matrix table of tomato fruit weight and different fruit quality traits.

	Firmness	Brix	% TTA	DM% low cluster end harvest	phenolics µmol /g FW
Fruit weight	NS	0.755	NS	0.722	NS
Firmness		-0.847	NS	-0.769	-0.718
Brix			NS	0.849	0.695
% TTA				NS	0.645
DM% low cluster end harvest					0.796

Table S8. Analysis of variance significance levels. Variables are abscisic acid (ABA), phaseic acid (PA), 9-hydroxy-ABA (9OH-ABA) indole-3-acetic acid (IAA), oxo-IAA (OxIAA), phenylacetic acid (PAA), salicylic acid (SA), benzoic acid (BzA), jasmonic acid (JA), and JA-isoleucine (JA-Ileu), trans-zeatin riboside (tZR), cis-zeatin-riboside (cZR), isopentenyl adenine-7-glucoside (iP7G), 1-aminocyclopropane-1-carboxylic acid (ACC). The data were analyzed using a 3-way-ANOVA. The first factor is top lighting at two levels (161 W m^{-2} and 242 W m^{-2}), the second factor is supplemental LED inter-lighting at two levels (no LED, 60 W m^{-2}), and the third factor is shoot branching at two levels (one- or two shoot plants) **(A)**. The data were also analyzed with a 2-factor ANOVA, where the first factor is supplemental LED inter-lighting (L) (without LED, 60 W m^{-2}, and 120 W m^{-2}), and the second factor is shoot branching (S) (one- or two-shoot plants) **(B)**. All treatments represent 4 or 5 replications. Means and SE are presented in Figure 9. Statistically significant effects or interactions between factors are indicated with asterisks, * *p*<0.05, ** *p*<0.01, *** *p*<0.001. NS[1] represents near statistical significance, with a probability between 0.05 and 0.066. The absence of a star denotes no significant difference at a significance level of *p*<0.05.

	**3-way ANOVA (A)**							**2-way ANOVA (B)**		
	Top light (T)	LED (L)	Shoot (S)	T x L	T x S	L x S	T x L x S	L	S	L x S
**Abscisic acid**										
ABA			**				*		*	
PA							*			
9OH-ABA									*	
**Auxins**										
IAA										
Ox-IAA									NS[1]	
PAA										
**Saliscylic acid, Benzoic acid**										
SA										
BzA										
**Jasmonic acid**										
JA			*						**	
JA_Ileu		*	*			*			*	
**Cytokinins**										
tZR			**						NS[1]	
cZR		**			*			*	*	
iP7G	NS[1]					**			*	
**Ethylene**										
ACC										

Table S9. Analysis of variance significance levels. The table presents the results of a 3-way ANOVA and 2-way ANOVA for variables of xylem sap exudation per root and shoot. In the 3-way ANOVA, the first factor is top lighting at two levels (161 W m^{-2} and 242 W m^{-2}) (T), the second factor is supplemental LED inter-lighting at two levels (no LED, 60 W m^{-2}) (L), and the third factor is shoot branching at two levels (one-shoot or two-shoot plants) (S) (A). The data were also analyzed by 2-factor ANOVA, with the first factor being supplemental LED inter-lighting at 161 W m^{-2} (without LED, 60 W m^{-2}, and 120 W m^{-2}) and the second factor being shoot branching (one-shoot or two-shoot plants) (B). The table includes results of statistical significance, with "***" indicating a significant effect (p < 0.001) of a factor. The absence of a star denotes no significant difference at a significance level of p<0.05.

		Xs/root	Xs/shoot
A)	**Toplight (T)**		
	**LED (L)**		
	**Shoot (S)**	***	
	**T x L**		
	**T x S**		
	**L x S**		
	**T x L x S**		
B)	**L**		
	**S**	***	
	**L x S**		

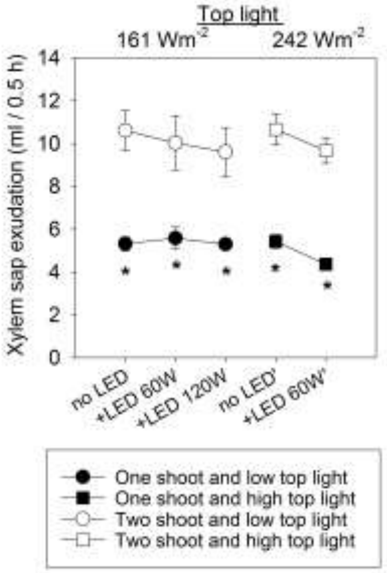

Figure S1. The impact of top light intensities, supplemental LED inter-lighting, and shoot branching on xylem sap exudation at final harvest. The figure presents mean values ± SE of xylem sap exudation in response to different treatments. The experimental factors studied were top lighting at two levels (161 W m^{-2} and 242 W m^{-2}), two or three levels of supplemental LED inter-lighting (no LED, +60 W m^{-2}, and +120 W m^{-2} for 161 W m^{-2} top light), and two levels of shoot branching (one- and two-shoot plants). The stars indicate statistically significant differences at $p < 0.05$ between one- and two-shoot plants within the same light treatment (LSD test) (n = 4-5).
